# Digital competence for sustainable education of pre-service teachers: a systematic literature review (2014–2024)

**DOI:** 10.3389/fpsyg.2025.1710983

**Published:** 2026-01-12

**Authors:** Lulu Zhang, Chunhong Yang, Youqi Zheng

**Affiliations:** Department of Foreign Languages, College of Liberal Arts, Nanjing University of Information Science and Technology, Nanjing, China

**Keywords:** assessment tools, digital competence, pre-service teachers, systematic review, training effectiveness

## Abstract

With the rapid advancement of educational digitalization, pre-service teachers’ digital competence has become a critical prerequisite for adapting to modern teaching practices and promoting high-quality education. This systematic review provides a comprehensive analysis of research on the digital competence of pre-service teachers over the past decade (2014–2024). Drawing on 38 studies retrieved from the Web of Science and Scopus databases, it examines several key aspects, including publication trends, frameworks, research themes and achievements, research methods, and current research limitations. Findings indicate a steady increase in scholarly publications on pre-service teachers’ digital competence over this period, reflecting the growing importance of digital literacy for effective teaching in modern classrooms. Among the frameworks employed, the Technological Pedagogical Content Knowledge (TPACK) framework emerges as the most widely adopted, helping researchers understand how technology can be effectively integrated into teaching practices. A notable trend is the integration of such frameworks, which enriches conceptual foundations and broadens practical applications. In addition, pre-service teachers generally demonstrate adequate digital skills for daily tasks, but show uneven performance across competence dimensions, with particular weaknesses in digital content creation, pedagogical integration, and problem-solving. The field has gradually evolved from descriptive analyses of competence levels toward more application-oriented inquiries, highlighting training design, assessment development, and technology integration. Review analysis reveals that pre-service teachers’ digital competence is influenced by a combination of demographic, psychological, educational, and socio-cultural factors. Several key limitations have been identified, including insufficient sample representativeness, less rigorous research designs, interference from background factors etc., underscoring the need for more robust research designs, diverse assessment instruments, and larger, more representative samples to strengthen the evidence base. The findings also reveal gaps in complex technical operations and teaching applications, as well as in training programs related to technology integration and innovation. By providing a comprehensive synthesis of existing literature, this review offers valuable insights for educators, policymakers, and researchers aiming to foster the digital readiness of future teachers. Future research should address these limitations by employing larger and more diverse samples, integrating mixed-methods designs, including multilingual and qualitative studies, and exploring digital competence in specific subjects or grade levels.

## 1 Introduction

With the rapid development of information technology, the education sector is undergoing a profound digital transformation. Digital teaching has become an important component of modern education (Horvath et al., 2025). Beyond transforming traditional teaching methods and learning models to make education more flexible, diverse, and efficient, this shift also introduces new challenges (Caena and Redecker, 2019). Amidst this transformative landscape, the global focus on education quality and equity resonates profoundly with the objectives outlined in United Nations (2015), particularly Sustainable Development Goal 4 (SDG 4), which underscores the significance of inclusive and equitable quality education and lifelong learning opportunities for all.

In this global context, pre-service teachers, as the main contributors to future education, need to possess sufficient digital capabilities to cope with the challenges brought about by technological changes and to better adapt to the development trends of modern education (Palacios-Rodriguez et al., 2023). Their digital proficiency plays a key role in driving the digital transformation of education while also making a direct contribution to achieving SDG 4, which is accomplished by improving educational quality, advancing equity, and encouraging lifelong learning. Mastery of digital technologies enables pre-service teachers to leverage digital teaching resources more effectively, innovate pedagogical methods, and consequently, improve teaching efficacy. Furthermore, it is crucial for educators to integrate technology into their classrooms (Balula et al., 2022). The digital ability of pre-service teachers influences both their own continuous professional development (Chu et al., 2023; Instituto Nacional de Tecnologías Educativas y de Formación del Profesorado (INTEF), 2017) and the learning outcomes of students, along with the cultivation of students’ digital literacy (López-Nuñez et al., 2024). In the digital age, students need to possess good digital literacy, including information acquisition, processing, analysis, and innovation abilities. Pre-service teachers can effectively guide students in using digital technology correctly, cultivate their digital thinking and innovation abilities, and lay a solid foundation for their future learning and work by enhancing their digital skills. Consequently, focusing on the digital competence of pre-service teachers is an essential aspect of the digital transformation of education and serves as a crucial strategy for enhancing the overall quality of education and the holistic development of students. Beyond access to technology, the psychological readiness of pre-service teachers merits equal attention. The confidence has a significant impact on pre service teachers’ willingness to try new resources and their persistence in the face of difficulties. Research shows that is essential for pre-service teachers to have both positive beliefs in information and digital skills to successfully integrate digital technology into the teaching environment (Sergeeva et al., 2023). Moreover, their attitude toward educational technology is reported to be an important factor influencing their future professional use of such tools (Janeš et al., 2023). A positive attitude appears to motivate pre-service teachers to actively explore digital technologies, seek continuous professional-development opportunities, and, consequently, attain higher levels of digital competence (Tondeur et al., 2018). Thus, confidence and positive attitudes are catalysts that convert technical access into classroom-ready innovation.

In the digital era, pre-service teachers’ digital competence has become a critical indicator of their overall professional readiness. Research indicates that high levels of digital literacy are essential for the sustainable development of pre-service teachers’ professional capabilities (Esteve-Mon et al., 2020). However, to date, the literature comprises only two systematic reviews that examine the digital competence of pre-service teachers: Tarraga-Minguez et al.’s (2021) review of Spanish pre-service teachers’ digital teaching competence and Yang et al.’s (2023) systematic review of digital competence among Chinese K-12 pre-service and in-service teachers. While informative, these reviews have significant limitations in scope. They primarily focus on specific countries or regions, such as Spain or China, lacking a comprehensive analysis of pre-service teachers’ digital competence on a global scale. This regional limitation restricts the generalizability of findings to other cultural and educational contexts, thereby hindering a deeper understanding of the commonalities and differences in the development of pre-service teachers’ digital competence. Furthermore, regarding the time frame, Tarraga-Minguez et al.’s study only covers studies from 2011 to 2020, while research on pre-service teachers’ digital competence has grown significantly in the past 5 years, particularly in quantitative studies. Although Yang et al. extended their search period to 2010–2023, their analysis focused on both pre- and in-service teachers, failing to provide an in-depth examination of pre-service teachers’ unique needs and challenges in developing digital teaching competencies. As they synthesize research from a specific past period, their conclusions may not fully address the rapidly evolving digital landscape, prompting calls for more current and comprehensive analyses.

Thus, this study conducts a systematic review of research on pre-service teachers’ digital competence from 2014 to 2024 to explore how this field has evolved over time. It seeks to identify the main trends, conceptual frameworks, and research focuses, as well as to examine the methodological characteristics and limitations of existing studies. Accordingly, the review is guided by the following research questions:

(1) What are the emerging trends, conceptual frameworks, and research focuses identified in studies on pre-service teachers’ digital competence?
(2) What methodological characteristics and limitations can be identified across these studies?

## 2 Digital competence frameworks

### 2.1 Definition

Digital competence is a multidimensional and dynamically evolving concept with diverse interpretations across policy documents, academic research, and teaching practices (Ferrari et al., 2012). Drawing on the core frameworks and definitions proposed by the Council of the European Union (2018), it can be defined as an individual’s ability to effectively, critically, and securely use information technology in digital environments, including the capacity to acquire, manage, understand, evaluate, create, and share digital information, as well as to communicate, collaborate, and solve problems using digital tools.

While various definitions exist for digital skills, digital abilities, and other related terms (Perifanou and Economides, 2019), digital abilities, and digital literacy have emerged as core concepts for describing digital proficiency. Some researchers contend that digital competence extends beyond digital literacy, encompassing a spectrum of capabilities from basic digital skills to advanced capacities such as critical thinking, digital security, and ethical technology use (Hammoda and Foli, 2024). This broader perspective positions digital competence as a more comprehensive and integrated concept (Yang, 2024). However, the terms “digital competence” and “digital literacy” are often used interchangeably in practice, particularly within the European context (Madsen et al., 2018).

### 2.2 Frameworks

To support the development of digital capabilities, researchers, international organizations, and governments various frameworks and standards have been developed. Within educational technology research, the Technological Pedagogical Content Knowledge (TPACK) framework (Mishra and Koehler, 2006) has been the subject of considerable scholarly inquiry and application. This framework builds upon Pedagogical Content Knowledge (PCK) model and emphasizes the transformation of subject content knowledge into a unique knowledge system for effective teaching. Mishra and Koehler expanded this model by incorporating the dimension of technological knowledge (TK), illustrating how the integration of technology, pedagogy, and content knowledge can generate diverse forms of teacher expertise.

The TPACK framework was designed to provide a conceptual model for teachers to effectively design and implement technology-enhanced learning (Mishra and Koehler, 2006; Mishra and Koehler, 2008). Today, TPACK has become a pivotal framework for describing and understanding the knowledge required for integrating technology into teacher education, particularly for pre-service teachers (Valtonen et al., 2020). Its influence on educational technology research and practice has been profound, especially in pre-service teacher training, where the TPACK framework has been widely adopted (Lachner et al., 2021).

In recent years, with the increasing integration of artificial intelligence (AI) in education, the importance of embedding AI applications into educational environments has become more pronounced. Consequently, assessing teachers’ abilities in AI-related Technological Pedagogical Content Knowledge (TPACK) has gained critical importance, leading to the emergence of the concept of AI-TPACK (Ning et al., 2024).

Since 2006, digital competence has been recognized by the European Union as one of the key competencies for lifelong learning (European Union, 2018), signifying the widespread acknowledgment of the importance of digital literacy in personal and societal development. To further advance this initiative, the European Union (2010) launched *A Digital Agenda for Europe* in May 2010, placing the enhancement of digital competence at its core and aiming to lead Europe into a fully digital era. This agenda identified digital technologies as a critical driver for sustainable development and proposed a series of concrete measures to establish a solid foundation for achieving sustainable development goals.

To address the rapid evolution of the digital society, the European Commission’s Joint Research Centre (JRC) released the first European Digital Competence Framework for Citizens (DigComp) in 2013 (Ferrari, 2013). Since then, the framework has undergone several updates from DigComp 2.0 in 2016 to DigComp 2.2 in 2022. These updates reflect the rapid advancement of digital technologies and the continuous refinement of the concept of digital competence. This framework aligns closely with the *European Skills Agenda for Sustainable Competitiveness, Social Fairness and Resilience* (European Union, 2020). DigComp serves as a tool for enhancing citizens’ digital competence (Różewski et al., 2021) and is closely linked to the Sustainable Development Goals (SDGs). For example, it emphasizes that teachers’ use of digital technologies to improve and innovate learning environments is significant for achieving SDG 4 (Quality Education) and SDG 10 (Reduced Inequalities) (Gisbert-Cervera et al., 2022).

Derived from the DigComp framework, the European Framework for the Digital Competence of Educators (DigCompEdu) was developed by the European Commission as a specialized competency model for educators (Redecker, 2017). The framework provides educators with a systematic tool for developing digital competence, addressing their growing need to identify specific digital skills for their profession and to integrate technological advancements into educational practices. It assists teachers in assessing and enhancing their digital competence (Santos, 2023), while also informing and guiding their classroom implementation and ongoing professional development (Caena and Redecker, 2019). Additionally, it serves as a reference for educational institutions in formulating digital education policies and training programs.

The DigCompEdu framework encompasses three overarching areas that cover various aspects of educators’ digital competence: Educators’ Professional Competencies, Educators’ Pedagogic Competences, and Learners’ Competences. These areas are further divided into six distinct subdomains, commonly known as competence areas: Professional Engagement, Digital Resources, Teaching and Learning, Assessment, Empowering Learners, and Facilitating Learners’ Digital Competence.

Since its introduction in 2017, the DigCompEdu framework has been widely adopted across the European Union, serving as a foundational model for many member states to develop tailored national standards for teachers’ digital competence. A notable example is Spain, where the National Institute of Educational Technologies and Teacher Training (INTEF) synthesized the core ideas of DigComp 2.0 and DigCompEdu to formulate the *Common Framework for Teachers’ Digital Competence* (2017). This framework systematically delineates 21 essential competencies for teachers, categorized into five core domains: information processing and literacy, collaboration and communication, digital content creation, information security, and problem-solving. It establishes clear benchmarks and pathways for enhancing digital competence among Spanish educators.

While the INTEF framework exhibits structural and conceptual differences from DigCompEdu—particularly in the latter’s broader focus on students’ and institutions’ digital competence and its more extensive competency dimensions (Instituto Nacional de Tecnologías Educativas y de Formación del Profesorado (INTEF), 2017)—the INTEF framework has significantly contributed to strengthening teachers’ digital teaching proficiency. It provides a robust theoretical and practical foundation for educators to navigate through and thrive in digital teaching environments.

## 3 Method

The PRISMA guidelines for conducting and reporting systematic reviews were adhered to during the review process.

### 3.1 Search strategy

Two high-quality academic databases—Web of Science (WoS) and Scopus—were selected for the literature search, following the procedures adopted in previous systematic review studies. The search terms were determined based on the research questions, and search strings were constructed using the Boolean operators OR and AND to ensure comprehensive coverage of relevant literature.

For WoS, the search query was formulated as:

(digital competence) OR (digital literacy) OR (digital skill) AND (pre-service teacher*) OR (future teacher*)*.

For Scopus, the query used was:

((“digital competence”) OR (“digital literacy”) OR (“digital skill”)) AND ((“pre-service teacher”) OR (“future teacher”)).

Through this process, a total of 989 documents were identified, including 524 from WoS and 465 from Scopus.

### 3.2 Document selection

#### 3.2.1 Inclusion and exclusion criteria

To ensure methodological rigor and enhance the comparability of results across diverse samples, this review focused on quantitative and mixed-method studies that demonstrated methodological transparency, internal validity, and generalizability (Arnkoff et al., 1996). Qualitative or case-study designs, which often yield context-dependent findings and face limitations in external validity (Crowe et al., 2011), were excluded to maintain analytic consistency. Mixed-method studies were retained if they contained a substantial quantitative component reporting measurable outcomes related to digital competence, aligning with the purpose of this review—to map large-scale trends and patterns rather than to conduct a qualitative thematic synthesis.

Specifically, the inclusion criteria required that studies provide empirical evidence and focus specifically on the digital competence of pre-service teachers. Eligible studies were expected to adopt quantitative or mixed-method approaches. Additionally, only studies published in English between 2014 and 2024 and fully accessible via institutional or affiliated association subscriptions were considered. Conversely, studies were excluded if they did not provide empirical evidence or were unrelated to digital competence. Studies focusing on university professors or in-service teachers, or those employing solely qualitative methods, were also excluded. Additionally, publications outside the 2014–2024 period, not written in English, inaccessible in full text, or presented as reports, reviews, conference papers, or book chapters were not considered for inclusion. After removing duplicates, 872 records remained. During the preliminary screening of titles, abstracts, and keywords, 112 studies (12% of the total) were retained. When titles and abstracts did not provide sufficient information, full texts were consulted for decision-making.

#### 3.2.2 Quality criteria

**Figure 1:**
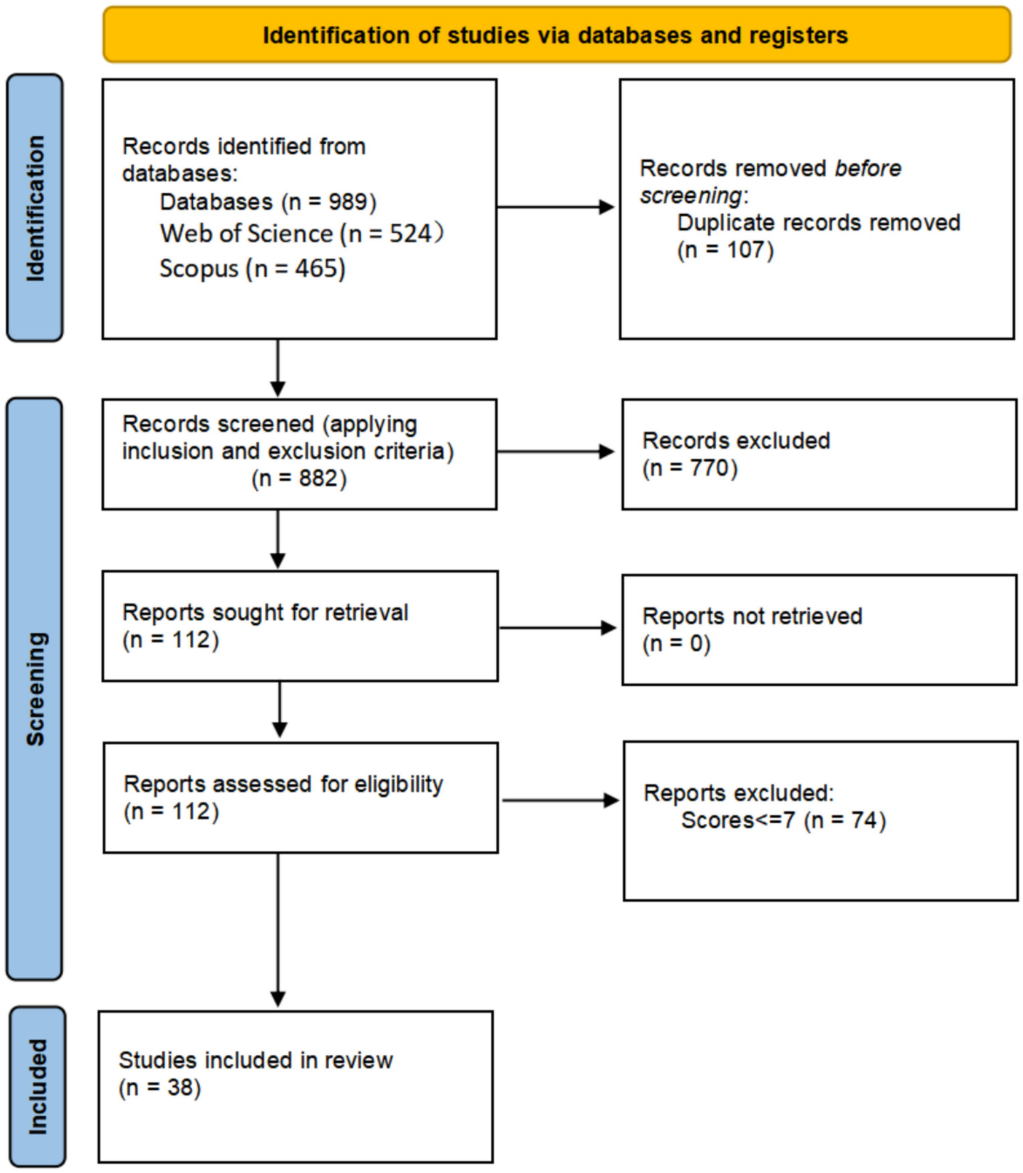
PRISMA flow diagram. Details of the number of studies selected and excluded at each stage, along with the reasons.

The 112 selected studies were further assessed using established quality criteria based on the research questions. First, it was assessed whether the concept of digital competence was clearly defined and whether the research objectives were clearly stated and effectively pursued. The studies were also examined to determine if the instruments were clearly described and design-based, and if the competence models and frameworks were properly presented. Furthermore, the assessment of digital competence dimensions and the measurement of pre-service teachers’ digital competence were considered. Finally, the studies were evaluated based on how well the research questions were addressed, whether the conclusions were clearly described and supported by the results, and whether the discussion adequately considered the research questions and limitations. Each criterion was scored as “yes” (1 point), “no” (0 points), or “partially” (0.5 points), allowing a total score between 0 and 10. Studies scoring 7.5 or higher were included in the final analysis. Applying these standards resulted in the exclusion of 74 studies, leaving 38 documents (34% of the considered articles) to address the research questions. The data extraction process is summarized in Figure 1 using the PRISMA 2020 flow diagram, showing the number of studies selected and excluded at each stage along with the corresponding reasons.

### 3.3 Review and coding process

In the fourth step, the selected articles were thoroughly read, and key information was extracted and organized in an Excel spreadsheet to address the research questions. To ensure consistency and reliability in coding, one author conducted the initial screening based on the predefined inclusion and exclusion criteria, while a second author independently reviewed these preliminary judgments. Any discrepancies were discussed until consensus was reached, and the 38 selected articles were then coded to facilitate systematic analysis.

## 4 Results

The following sections systematically presents the results in relation to the research questions. For detailed verification of each included study’s attributes, methodological details, and core content, refer to the supporting tables: Table 1 provides comprehensive information (e.g., Study, Year, Country, Sample, Conceptual Framework, Key Findings etc.); Tables 2–4 further categorize the articles by research focuses, influencing factors, and continents, ensuring research transparency and facilitating in-depth exploration.

**Table 1:** Summary of the selected articles.

Study	Year	Country	Sample	Design	Topic	Key findings	Conceptual framework	Instrument	Instrument validation	Influencing factors	Limitations
Çebi et al. (2022)	2022	Turkey	24 pre-service teachers	Mixed method	To analyze pre-service teachers’ progress in digital competence and TPACK after training; to investigate the digital knowledge and skills used in technology integration; to explore correlations between digital competences and TPACK.	Pre-service teachers showed significant improvement in the five areas of DigComp, and the sub-dimensions of design, exertion, ethics, and proficiency after the intervention.	DigComp and TPACK-deep Frameworks	Questionnaire with Likert-type questions Participant diary Focus-group interview	Cronbach’s *α*	None	Data Collection Tools, Research Design
Maiier and Koval (2021)	2021	Ukraine	56 pre-service FL teachers	Mixed method	To assess pre-service FL teachers’ readiness to use digital resources, attitudes toward digital competence development, and outline development approaches.	Pre-service foreign-language teachers who used a digital-skills checklist and received regular teacher guidance improved their digital competence significantly more than those who were only consulted by their teachers.	General Framework	Checklists Questionnaire	None	Teaching strategy	Sample Representativeness
Hijón-Neira et al. (2023)	2023	Spain	116 pre-service teachers	Quantitative	To test the effect of implementing a digital competence framework in pre-service teacher training; improve their overall digital competence and programming knowledge.	Pre-service teachers significantly improved in digital competence and programming knowledge after the intervention.	INTEF Framework	Questionnaire	Cronbach’s *α*	None	None
Andreasen et al. (2022)	2022	Norway	128 and 118 pre-service teachers	Quantitative	To measure pre-service teachers’ professional digital competence (PDC) before and after teacher education reform; to explore gender differences in ICT self-efficacy; to assess their perception of teacher educators’ PDC needs.	Pre-service teachers showed significant increase in digital competence after teacher education reform. Gender differences were found in ICT self-efficacy.	Self-Designed Frameworks Based on Literature Reviews	Questionnaire	Cronbach’s *α*	Gender National teacher education reform	Data Collection Tools, Research Content/Variables, Research Design
Miguel-Revilla et al. (2020)	2020	Spain	50 pre-service teachers	Quantitative	To measure pre-service social studies teachers’ TPACK competence and assess the effectiveness of a project-based learning intervention.	Pre-service teachers had low initial levels of TPACK, but improved significantly after the intervention.	TPACK Framework	Questionnaire with Likert-type questions	Cronbach’s *α*	None	Sample Representativeness Data Collection Tools Research Design
Al-Abdullatif (2019)	2019	Saudi Arabia	113 pre-service teachers	Quantitative	To assess pre-service teachers’ technological knowledge (TK) and TPACK confidence; to identify gender and age differences in TK and TPACK confidence; to evaluate the effectiveness of teacher education programs in fostering TPACK.	Pre-service teachers had very low levels of technological knowledge and TPACK confidence. Female pre-service teachers showed significantly greater confidence and readiness to use ICT for transforming student learning.	TPACK Framework	Questionnaire with Likert-type questions	Cronbach’s *α*	Gender Age	Sample Representativeness
Rodríguez-García et al. (2022)	2022	Spain	698 pre-service teachers	Quantitative	To analyze pre-service teachers’ self-perception of digital competence in communication and collaboration; to explore gender differences in this competence area.	Pre-service teachers had an intermediate level of digital competence for communication and collaboration. Gender differences were found.	DigComp Framework	Likert Scale	Cronbach’s *α*, KMO, Bartlett	Gender	Sample Representativeness, Data Collection Tools
Guillen-Gamez et al. (2020)	2020	Spain	675 pre-service teachers	Quantitative	To evaluate pre-service teachers’ digital competence (attitude, knowledge, use) and compare differences by educational modality (face-to-face vs. blended) and gender.	Pre-service teachers had a medium level of digital competence. No gender differences, but gender differences were found between face-to-face and blended learning modalities.	Self-Designed Frameworks Based on Literature Reviews	Questionnaire with Likert-type questions	Cronbach’s *α*	Gender Education model Attitude	Sample Representativeness Data Collection Tools Research Content/Variables
Palacios-Hidalgo and Huertas-Abril (2022)	2022	Spain	126 pre-service EFL teachers	Quantitative	To examine pre-service EFL teachers’ self-perceived digital literacy; to identify gender and academic year differences in digital competence perceptions; to assess their knowledge of digital competence frameworks.	Pre-service EFL teachers positively self-perceived their digital competence, but felt they did not work enough on it during their university degree. Lower-year students scored higher, and men consistently outperformed women.	Conceptual Frameworks Developed by Other Authors	Questionnaire	Cronbach’s *α*	Gender Degree/grade	Sample Representativeness, Data Collection Tools, Research Content/Variables
Çebi and Reisoğlu (2020)	2020	Turkey	518 pre-service teachers	Quantitative	To assess pre-service teachers’ digital competence and explore its variations by gender, branch, and perceived digital competence level.	Pre-service teachers had moderate digital competence, with lower levels in content creation and problem-solving. Gender, branch were factors.	DigComp Framework	Questionnaire with Likert-type questions	None	Gender Discipline branch	Research Content/Variables
Galindo-Domínguez and Bezanilla (2021)	2021	Spain	200 pre-service teachers	Quantitative	To investigate pre-service teachers’ digital competence profile, and explore differences by degree, gender, course, and age.	Pre-service teachers had a medium level of digital competence, with difficulties in the content creation dimension. Digital competence improved over the years of the degree. No significant differences were found regarding type of university and gender	INTEF Framework	Questionnaire with Likert-type questions	Cronbach’s *α*	Gender Age Degree/Grade	Sample Representativeness Data Collection Tools Research Design Research Context/Environment
Cañete et al. (2022)	2022	Paraguay	330 pre-service teachers	Quantitative	To assess final-year pre-service teachers’ self-perceived digital competence across four dimensions; to identify differences by gender, age, ICT training and frequency of use.	Preservice teachers had very basic to basic perception about their digital competence. Competence was related to gender, frequency of ICT use, and ICT training. No age differences.	Self-Designed Frameworks Based on Literature Reviews	Likert Scale	Cronbach’s *α*	Gender Age Usage frequency	Sample Representativeness, Data Collection Tools Research Design
Alnasib (2023)	2023	Saudi Arabia	140 pre-service teachers	Mixed method	To assess pre-service teachers’ self-perceived digital competence; evaluate whether teacher preparation programs qualify them for digital education.	Majority of pre-service teachers rated their digital competence as excellent, but felt their preparation program was only moderately effective in qualifying them for digital education.	DigComp Framework	Questionnaire with both closed-ended and open-ended questions	Cronbach’s *α*	Attitude	Sample Representativeness Data Collection Tools Research Content/Variables
Jiménez-Hernández et al. (2020)	2020	Spain	485 pre-service teachers	Quantitative	To measure pre-service secondary school teachers’ digital competence and analyze differences by gender, age (Z-Generation), and branch of knowledge.	Pre-service teachers showed homogeneous digital competence levels, but differences were found in specific areas based on gender, age, and subject area.	DigCompEdu Framework	Questionnaire with Likert-type questions	Cronbach’s *α*	Gender Age Discipline branch	Data Collection Tools
Hairida et al. (2023)	2023	Indonesia	885 pre-service	Quantitative	To validate the adapted digital literacy questionnaire; investigate pre-service chemistry teachers’ digital literacy ability.	The study validated a three-dimensional digital literacy scale for Indonesian preservice chemistry teachers Found them strongest in technical skills, notably weaker in cognitive and social–emotional domains, with no meaningful gender gaps.	Conceptual Frameworks Developed by Other Authors	Questionnaire with Likert-type questions	Cronbach’s *α* Rasch analysis	Gender	Sample Representativeness, Research Design, Research Content/Variables
Johnson et al. (2024)	2024	Germany	242 pre-service teachers	Mixed method	To evaluate the effectiveness of an extracurricular training program on pre-service teachers’ DigCompEdu competences; to assess changes in their attitudes, competence beliefs, and test-based competences.	Pre-service teachers showed improvement only in confidence using digital technologies for subject-specific purposes after the training program, guiding future curricular refinements.	TPACK Framework	Likert Scale Knowledge tests Interview	Cronbach’s *α*	Teaching strategy	Sample Representativeness Data Collection Tools Research Context/Environment
Kukul (2022)	2022	Turkey	29 pre-service ICT teachers	Mixed method	To evaluate the impact of digital storytelling on pre-service ICT teachers’ perceived TPACK levels; to assess changes in their teaching proficiency self-efficacy; to explore their views on the digital storytelling process.	Digital storytelling significantly improved pre-service ICT teachers’ TPACK and teaching self-efficacy levels.	TPACK Framework	Likert Scale Open-ended interview questions	None	None	Sample Representativeness, Data Collection Tools, Research Design
Sergeeva et al. (2023)	2023	Russia	324 pre-service teachers	Quantitative (SEM)	To explore the impact of communication skills (COMP, SELF, SCC, LI-S) on pre-service teachers’ ICT competencies (ICTC-SS, ICTC-ID).	Pre-service teachers’ perceived COMP in communication skills positively influenced their information and communication technology competencies (ICTCs) for student support and instructional design.	General Framework	Likert Scale	Cronbach’s *α*	Gender Discipline branch Communication skills	Sample Representativeness Data Collection Tools Research Content/Variables
Polly et al. (2023)	2023	USA	105 pre-service and in-service teachers	Quantitative	To examine pre-service and in-service teachers’ perceptions of digital technologies’ importance, helpfulness, competence, and interest; explore desired supports.	Pre-service teachers rated digital technologies as more important, helpful, and themselves as more competent compared to in-service teachers. Preferences for learning supports also differed.	Self-Designed Frameworks Based on Literature Reviews	Questionnaire with Likert-type questions	Cronbach’s *α*	None	Sample Representativeness Data Collection Tools Research Content/Variables
Yang et al. (2022)	2022	China	250 pre-service, 248 in-service teachers	Quantitative	To measure pre-service and in-service teachers’ perceived digital competence; to explore socio-demographic factors influencing this competence; to compare digital competence between the two teacher groups.	Although ICT awareness is strong, teaching practice remains weak. In-service teachers’ digital competence exceeds that of pre-service peers and correlates significantly with age, teaching years, and educational background.	Frameworks Based on National Standards	Likert Scale	Cronbach’s *α* CFA	Gender Age Degree/grade	Sample Representativeness, Data Collection Tools
McGarr and McDonagh (2021)	2021	Ireland	208 pre-service teachers	Mixed method	To explore pre-service teachers’ digital competence on entry into initial teacher education, including technical skills, cyber ethics knowledge, and attitudes toward technology in teaching.	Pre-service teachers were active technology users, but reported lower skills in using other digital technologies. Their knowledge of cyber ethics and associated practices also varied.	Conceptual Frameworks Developed by Other Authors	Questionnaire with both open-ended and Likert-type questions	None	None	Data Collection Tools Research Content/Variables
Sergeeva et al. (2024)	2024	Russia	163 pre-service teachers	Quantitative	To explore pre-service teachers’ ICT competence beliefs; to identify gender and grade level differences in ICT competence beliefs.	Pre-service teachers had good ICT competence beliefs, with gender differences in a few dimensions and no grade differences. Lower scores were found in analyzing, reflecting, problem-solving, and information/data literacy.	Conceptual Frameworks Developed by Other Authors	Questionnaire with Likert-type questions	Cronbach’s *α*	Gender Degree/grade	Sample Representativeness Data Collection Tools Research Content/Variables
Hirsch and Rubach (2024)	2024	Germany	308 pre-service teachers	Quantitative	To examine the changes in pre-service teachers’ TPK and TPACK over one semester; to evaluate the impact of SQD teaching strategies on the development of TPK and TPACK.	No significant overall change in pre-service teachers’ TPK and TPACK across seminars. The orchestration of SQD teaching strategies positively impacted the development of TPK and TPACK.	TPACK Framework	Likert Scale	Cronbach’s *α* EFA CFA	Teaching strategy	Sample Representativeness Data Collection Tools Research Design Research Content/Variables
Belda-Medina and Calvo-Ferrer (2022)	2022	Spain	85 pre-service teachers	Mixed method	To assess pre-service teachers’ digital competence and pedagogical skills for AR integration in CLIL classrooms; to analyze their attitudes toward AR applications; to explore correlations between TPACK and AR attitudes.	Pre-service teachers lacked practical knowledge on AR content creation and implementation, especially in the TPK intersection.	TPACK Framework	Likert Scale Semi-structured focus group discussions	Cronbach’s *α*	None	Sample Representativeness, Research Context/Environment, Data Collection Tools
Xu et al. (2019)	2019	China	905 pre-service teachers	Quantitative	To examine the relationship between pre-service teachers’ interpersonal communication competence (ICC) and digital citizenship; to identify gender differences in ICC and digital citizenship; to explore the predictive power of 10 ICC skills on digital citizenship.	Interpersonal communication competence skills positively predicted digital citizenship among pre-service teachers.	General Framework	Questionnaire with Likert-type questions	Cronbach’s *α*	Gender Communication skills	Sample Representativeness Data Collection Tools Research Content/Variables
Nurzhanova et al. (2024)	2024	Kazakhstan	209 pre-service teachers	Quantitative	To examine pre-service teachers’ digital literacy and technology use skills; to identify differences by gender and grade level; to explore the predictive role of digital literacy on technology use skills.	Pre-service teachers had high levels of technology use skills and digital literacy, with differences by gender and grade level.	General Framework	Questionnaire with Likert-type questions	Cronbach’s *α*	Gender Degree/grade	None
Yurdakul	2017	Turkey	1,493 pre-service teachers	Quantitative (SEM)	To explore the relationship between pre-service teachers’ digital nativity and TPACK competencies; to verify whether digital nativity predicts TPACK competencies.	Digital nativity was a significant predictor of pre-service teachers’ TPACK competency.	TPACK-deep Framework	Likert Scale	Cronbach’s *α* EFA CFA	Digital nativity	Sample Representativeness Data Collection Tools Research Content/Variables
Luik et al. (2018)	2018	Estonia	413 pre-service teachers	Quantitative	To validate a general TPACK measurement instrument in the Estonian context; to describe pre-service teachers’ perceptions of TPACK; to explore relationships between TPACK perceptions and gender, age, and study level.	Pre-service teachers lacked pedagogical knowledge, but perceived they were good at integrating technology into teaching. Differences were found by gender, age, and curricula.	TPACK Framework	Questionnaire with Likert-type questions	Cronbach’s *α* EFA CFA	Gender Age Degree/grade	Sample Representativeness Data Collection Tools
Janeš et al. (2023)	2023	6 countries (Norway, Slovenia, Portugal, Turkey, Ukraine and Jordan)	573 pre-service teachers	Mixed method	To investigate factors influencing pre-service teachers’ future use of digital technologies; assess their attitudes, knowledge, and skills toward DT.	Knowledge, skills, and attitudes toward digital technology were significant predictors of pre-service teachers’ future professional use of digital technology.	Self-Designed Frameworks Based on Literature Reviews	Questionnaire (including open-ended, optional questions and Likert scale)	Cronbach’s *α*	Attitude National/regional differences	Data Collection Tools
Peled (2021)	2021	Israel	1,265 pre-service teachers	Quantitative	To assess pre-service teachers’ self-perceived digital literacy levels and digital readiness, and explore the predictive effect of background characteristics.	Over 50% of pre-service teachers reported high digital literacy levels. Their sense of readiness for teamwork and ethics was high, but lower in first and advanced order of readiness.	Conceptual Frameworks Developed by Other Authors	Questionnaire with Likert-type questions	Cronbach’s *α*	Degree/Grade	Sample Representativeness Data Collection Tools
Quast et al. (2023)	2023	Germany	1386 (prospective) teachers	Quantitative	To develop and validate an instrument to assess professional digital competence beliefs of student teachers, pre-service teachers and teachers; compare group differences.	Seven dimensions of professional digital competence beliefs were identified, with pre-service teachers holding the highest beliefs, followed by in-service teachers.	DigCompEdu Framework	Questionnaire with Likert-type questions	Cronbach’s *α* EFA CFA	Age	Sample Representativeness, Data Collection Tools, Research Context/Environment
Rahimi and Mosalli (2024)	2024	Iran	472 pre-service EFL teachers	Quantitative (PLS-SEM)	To explore the role of pre-service EFL teachers’ 21st-century digital competence in shaping their 21st-century digital skills; to validate the DIGIGLO framework in the Iranian EFL context.	Four areas of digital global competence (DIGIGLO) shaped pre-service EFL teachers’ 21st century digital skills, including critical thinking.	Conceptual Frameworks Developed by Other Authors	Questionnaire	Cronbach’s *α*	None	None
Llanes (2023)	2023	Spain	20 pre-service teachers	Mixed method	To explore pre-service teachers’ perceptions of whether their training program and internship enabled them to develop digital teaching competence.	Pre-service teachers felt their training program did not adequately develop their digital teaching competence. More attention is needed to knowledge, skills, and attitudes in education for digital safety.	DigComp Framework	Questionnaire Semi-structured interviews	None	None	None
Lim (2023)	2023	USA	212 pre-service early childhood teachers	Quantitative	To explore the effects of pre-service teachers’ digital literacy and self-efficacy on their perception of AI education for young children.	Pre-service teachers had high critical thinking but low problem-solving in digital literacy. Their digital literacy and self-efficacy positively influenced their perception of AI education for young children.	General Framework	Questionnaire with Likert-type questions	Cronbach’s *α*	Self-efficacy	Sample Representativeness Data Collection Tools
Romero-Tena et al. (2020)	2020	Spain	535 pre-service early childhood teachers	Quantitative	To investigate pre-service early childhood teachers’ self-perceived digital competence before and after ICT training; to determine whether there are significant differences in self-perception after training; to identify pre-service teachers’ digital competence profiles.	Pre-service teachers’ self-perception of digital competence improved after receiving training, with shifts from lower to higher competence profiles.	Self-Designed Frameworks Based on Literature Reviews	Questionnaire with Likert-type questions	Cronbach’s *α*	Usage frequency	Sample Representativeness
Torres-Hernández and Gallego-Arrufat (2024)	2024	Spain	1,366 pre-service teachers	Mixed method	To examine pre-service teachers’ self-perceived competences in digital safety; to analyze the differences in digital safety perceptions by gender, age, and degree program.	Pre-service teachers had an intermediate level of digital safety competence. Gender, age, and degree program were differentiating factors in some dimensions. More attention is needed to digital safety education.	Frameworks Based on National Standards	Questionnaire (including dichotomous, open, Likert-type, multiple choice)	Cronbach’s *α* EFA CFA	Gender Age Degree/grade	Data Collection Tools Research Content/Variables
Gisbert-Cervera et al. (2022)	2022	Spain	1,350 pre-service teachers	Quantitative	To assess pre-service teachers’ self-perceived and objective TDC levels; to compare TDC scores between trained and untrained groups; to verify the effectiveness of specific TDC training.	Pre-service teachers who received specific digital competence training scored better than those without training.	Frameworks Based on National Standards	Questionnaire Assessment test	None	Degree/grade	Sample Representativeness
Ata and Yildirim (2019)	2019	Turkey	291 pre-service teachers	Mixed method	To explore pre-service teachers’ perceptions of digital citizenship; to identify differences in digital citizenship perceptions by gender, major, age, parental education level, and internet use; to collect pre-service teachers’ opinions on digital citizenship.	Male pre-service teachers significantly outscored females in digital citizenship, whereas department, high-school type and mother’s education showed no effect Only father’s education mattered. Participants felt proficient in digital communication and participation, credited parents partly, but deemed their major irrelevant.	General Framework	Questionnaire with both open-ended and Likert-type questions	Cronbach’s *α* EFA	Gender Age Discipline branch Usage frequency Parental education level	Sample Representativeness Data Collection Tools Research Content/Variables

**Table 2:** Overview of studies grouped by research focus.

Study	Year	Country	Sample	Design	Topic	Key findings	Conceptual framework	Instrument	Instrument validation	Influencing factors	Limitations
Current status and factors influencing digital competence											
Maiier and Koval (2021)	2021	Ukraine	56 pre-service FL teachers	Mixed method	To assess pre-service FL teachers’ readiness to use digital resources, attitudes toward digital competence development, and outline development approaches.	Pre-service foreign-language teachers who used a digital-skills checklist and received regular teacher guidance improved their digital competence significantly more than those who were only consulted by their teachers.	General Framework	Checklists Questionnaire	None	Teaching strategy	Sample Representativeness
Sergeeva et al. (2024)	2024	Russia	163 pre-service teachers	Quantitative	To explore pre-service teachers’ ICT competence beliefs; to identify gender and grade level differences in ICT competence beliefs.	Pre-service teachers had good ICT competence beliefs, with gender differences in a few dimensions and no grade differences. Lower scores were found in analyzing, reflecting, problem-solving, and information/data literacy.	Conceptual Frameworks Developed by Other Authors	Questionnaire with Likert-type questions	Cronbach’s *α*	Gender Degree/grade	Sample Representativeness Data Collection Tools Research Content/Variables
Çebi and Reisoğlu (2020)	2020	Turkey	518 pre-service teachers	Quantitative	To assess pre-service teachers’ digital competence and explore its variations by gender, branch, and perceived digital competence level.	Pre-service teachers had moderate digital competence, with lower levels in content creation and problem-solving. Gender, branch were factors.	DigComp Framework	Questionnaire with Likert-type questions	None	Gender Discipline branch	Research Content/Variables
Janeš et al. (2023)	2023	6 countries (Norway, Slovenia, Portugal, Turkey, Ukraine and Jordan)	573 pre-service teachers	Mixed method	To investigate factors influencing pre-service teachers’ future use of digital technologies; assess their attitudes, knowledge, and skills toward DT.	Knowledge, skills, and attitudes toward digital technology were significant predictors of pre-service teachers’ future professional use of digital technology.	Self-Designed Frameworks Based on Literature Reviews	Questionnaire (including open-ended, optional questions and Likert scale)	Cronbach’s *α*	Attitude National/regional differences	Data Collection Tools
Rodríguez-García et al. (2022)	2022	Spain	698 pre-service teachers	Quantitative	To analyze pre-service teachers’ self-perception of digital competence in communication and collaboration; to explore gender differences in this competence area.	Pre-service teachers had an intermediate level of digital competence for communication and collaboration. Gender differences were found.	DigComp Framework	Likert Scale	Cronbach’s *α*, KMO, Bartlett	Gender	Sample Representativeness, Data Collection Tools
McGarr and McDonagh (2021)	2021	Ireland	208 pre-service teachers	Mixed method	To explore pre-service teachers’ digital competence on entry into initial teacher education, including technical skills, cyber ethics knowledge, and attitudes toward technology in teaching.	Pre-service teachers were active technology users, but reported lower skills in using other digital technologies. Their knowledge of cyber ethics and associated practices also varied.	Conceptual Frameworks Developed by Other Authors	Questionnaire with both open-ended and Likert-type questions	None	None	Data Collection Tools Research Content/Variables
Torres-Hernández and Gallego-Arrufat (2024)	2024	Spain	1,366 pre-service teachers	Mixed method	To examine pre-service teachers’ self-perceived competences in digital safety; to analyze the differences in digital safety perceptions by gender, age, and degree program.	Pre-service teachers had an intermediate level of digital safety competence. Gender, age, and degree program were differentiating factors in some dimensions. More attention is needed to digital safety education.	Frameworks Based on National Standards	Questionnaire (including dichotomous, open, Likert-type, multiple choice)	Cronbach’s *α* EFA CFA	Gender Age Degree/grade	Data Collection Tools Research Content/Variables
Peled (2021)	2021	Israel	1,265 pre-service teachers	Quantitative	To assess pre-service teachers’ self-perceived digital literacy levels and digital readiness, and explore the predictive effect of background characteristics.	Over 50% of pre-service teachers reported high digital literacy levels. Their sense of readiness for teamwork and ethics was high, but lower in first and advanced order of readiness.	Conceptual Frameworks Developed by Other Authors	Questionnaire with Likert-type questions	Cronbach’s *α*	Degree/Grade	Sample Representativeness Data Collection Tools
Palacios-Hidalgo and Huertas-Abril (2022)	2022	Spain	126 pre-service EFL teachers	Quantitative	To examine pre-service EFL teachers’ self-perceived digital literacy; to identify gender and academic year differences in digital competence perceptions; to assess their knowledge of digital competence frameworks.	Pre-service EFL teachers positively self-perceived their digital competence, but felt they did not work enough on it during their university degree. Lower-year students scored higher, and men consistently outperformed women.	Conceptual Frameworks Developed by Other Authors	Questionnaire	Cronbach’s *α*	Gender Degree/grade	Sample Representativeness, Data Collection Tools, Research Content/Variables
Ata and Yildirim (2019)	2019	Turkey	291 pre-service teachers	Mixed method	To explore pre-service teachers’ perceptions of digital citizenship; to identify differences in digital citizenship perceptions by gender, major, age, parental education level, and internet use; to collect pre-service teachers’ opinions on digital citizenship.	Male pre-service teachers significantly outscored females in digital citizenship, whereas department, high-school type and mother’s education showed no effect Only father’s education mattered. Participants felt proficient in digital communication and participation, credited parents partly, but deemed their major irrelevant.	General Framework	Questionnaire with both open-ended and Likert-type questions	Cronbach’s *α* EFA	Gender Age Discipline branch Usage frequency Parental education level	Sample Representativeness Data Collection Tools Research Content/Variables
Alnasib (2023)	2023	Saudi Arabia	140 pre-service teachers	Mixed method	To assess pre-service teachers’ self-perceived digital competence; evaluate whether teacher preparation programs qualify them for digital education.	Majority of pre-service teachers rated their digital competence as excellent, but felt their preparation program was only moderately effective in qualifying them for digital education.	DigComp Framework	Questionnaire with both closed-ended and open-ended questions	Cronbach’s *α*	Attitude	Sample Representativeness Data Collection Tools Research Content/Variables
Galindo-Domínguez and Bezanilla (2021)	2021	Spain	200 pre-service teachers	Quantitative	To investigate pre-service teachers’ digital competence profile, and explore differences by degree, gender, course, and age.	Pre-service teachers had a medium level of digital competence, with difficulties in the content creation dimension. Digital competence improved over the years of the degree. No significant differences were found regarding type of university and gender	INTEF Framework	Questionnaire with Likert-type questions	Cronbach’s *α*	Gender Age Degree/Grade	Sample Representativeness Data Collection Tools Research Design Research Context/Environment
Cañete et al. (2022)	2022	Paraguay	330 pre-service teachers	Quantitative	To assess final-year pre-service teachers’ self-perceived digital competence across four dimensions; to identify differences by gender, age, ICT training and frequency of use.	Preservice teachers had very basic to basic perception about their digital competence. Competence was related to gender, frequency of ICT use, and ICT training. No age differences.	Self-Designed Frameworks Based on Literature Reviews	Likert Scale	Cronbach’s *α*	Gender Age Usage frequency	Sample Representativeness, Data Collection Tools Research Design
Nurzhanova et al. (2024)	2024	Kazakhstan	209 pre-service teachers	Quantitative	To examine pre-service teachers’ digital literacy and technology use skills; to identify differences by gender and grade level; to explore the predictive role of digital literacy on technology use skills.	Pre-service teachers had high levels of technology use skills and digital literacy, with differences by gender and grade level.	General Framework	Questionnaire with Likert-type questions	Cronbach’s *α*	Gender Degree/grade	None
Andreasen et al. (2022)	2022	Norway	128 and 118 pre-service teachers	Quantitative	To measure pre-service teachers’ professional digital competence (PDC) before and after teacher education reform; to explore gender differences in ICT self-efficacy; to assess their perception of teacher educators’ PDC needs.	Pre-service teachers showed significant increase in digital competence after teacher education reform. Gender differences were found in ICT self-efficacy.	Self-Designed Frameworks Based on Literature Reviews	Questionnaire	Cronbach’s *α*	Gender National teacher education reform	Data Collection Tools, Research Content/Variables, Research Design
Yang et al. (2022)	2022	China	250 pre-service, 248 in-service teachers	Quantitative	To measure pre-service and in-service teachers’ perceived digital competence; to explore socio-demographic factors influencing this competence; to compare digital competence between the two teacher groups.	Although ICT awareness is strong, teaching practice remains weak. In-service teachers’ digital competence exceeds that of pre-service peers and correlates significantly with age, teaching years, and educational background.	Frameworks Based on National Standards	Likert Scale	Cronbach’s *α* CFA	Gender Age Degree/grade	Sample Representativeness, Data Collection Tools
Al-Abdullatif (2019)	2019	Saudi Arabia	113 pre-service teachers	Quantitative	To assess pre-service teachers’ technological knowledge (TK) and TPACK confidence; to identify gender and age differences in TK and TPACK confidence; to evaluate the effectiveness of teacher education programs in fostering TPACK.	Pre-service teachers had very low levels of technological knowledge and TPACK confidence. Female pre-service teachers showed significantly greater confidence and readiness to use ICT for transforming student learning.	TPACK Framework	Questionnaire with Likert-type questions	Cronbach’s *α*	Gender Age	Sample Representativeness
Xu et al. (2019)	2019	China	905 pre-service teachers	Quantitative	To examine the relationship between pre-service teachers’ interpersonal communication competence (ICC) and digital citizenship; to identify gender differences in ICC and digital citizenship; to explore the predictive power of 10 ICC skills on digital citizenship.	Interpersonal communication competence skills positively predicted digital citizenship among pre-service teachers.	General Framework	Questionnaire with Likert-type questions	Cronbach’s *α*	Gender Communication skills	Sample Representativeness Data Collection Tools Research Content/Variables
Sergeeva et al. (2023)	2023	Russia	324 pre-service teachers	Quantitative (SEM)	To explore the impact of communication skills (COMP, SELF, SCC, LI-S) on pre-service teachers’ ICT competencies (ICTC-SS, ICTC-ID).	Pre-service teachers’ perceived COMP in communication skills positively influenced their information and communication technology competencies (ICTCs) for student support and instructional design.	General Framework	Likert Scale	Cronbach’s *α*	Gender Discipline branch Communication skills	Sample Representativeness Data Collection Tools Research Content/Variables
Hirsch and Rubach (2024)	2024	Germany	308 pre-service teachers	Quantitative	To examine the changes in pre-service teachers’ TPK and TPACK over one semester; to evaluate the impact of SQD teaching strategies on the development of TPK and TPACK.	No significant overall change in pre-service teachers’ TPK and TPACK across seminars. The orchestration of SQD teaching strategies positively impacted the development of TPK and TPACK.	TPACK Framework	Likert Scale	Cronbach’s *α* EFA CFA	Teaching strategy	Sample Representativeness Data Collection Tools Research Design Research Content/Variables
Guillen-Gamez et al. (2020)	2020	Spain	675 pre-service teachers	Quantitative	To evaluate pre-service teachers’ digital competence (attitude, knowledge, use) and compare differences by educational modality (face-to-face vs. blended) and gender.	Pre-service teachers had a medium level of digital competence. No gender differences, but gender differences were found between face-to-face and blended learning modalities.	Self-Designed Frameworks Based on Literature Reviews	Questionnaire with Likert-type questions	Cronbach’s *α*	Gender Education model Attitude	Sample Representativeness Data Collection Tools Research Content/Variables
Yurdakul (2018)	2017	Turkey	1,493 pre-service teachers	Quantitative (SEM)	To explore the relationship between pre-service teachers’ digital nativity and TPACK competencies; to verify whether digital nativity predicts TPACK competencies.	Digital nativity was a significant predictor of pre-service teachers’ TPACK competency.	TPACK-deep Framework	Likert Scale	Cronbach’s *α* EFA CFA	Digital nativity	Sample Representativeness Data Collection Tools Research Content/Variables
Lim (2023)	2023	USA	212 pre-service early childhood teachers	Quantitative	To explore the effects of pre-service teachers’ digital literacy and self-efficacy on their perception of AI education for young children.	Pre-service teachers had high critical thinking but low problem-solving in digital literacy. Their digital literacy and self-efficacy positively influenced their perception of AI education for young children.	General Framework	Questionnaire with Likert-type questions	Cronbach’s *α*	Self-efficacy	Sample Representativeness Data Collection Tools
Jiménez-Hernández et al. (2020)	2020	Spain	485 pre-service teachers	Quantitative	To measure pre-service secondary school teachers’ digital competence and analyze differences by gender, age (Z-Generation), and branch of knowledge.	Pre-service teachers showed homogeneous digital competence levels, but differences were found in specific areas based on gender, age, and subject area.	DigCompEdu Framework	Questionnaire with Likert-type questions	Cronbach’s *α*	Gender Age Discipline branch	Data Collection Tools
Training effectiveness											
Hijón-Neira et al. (2023)	2023	Spain	116 pre-service teachers	Quantitative	To test the effect of implementing a digital competence framework in pre-service teacher training; improve their overall digital competence and programming knowledge.	Pre-service teachers significantly improved in digital competence and programming knowledge after the intervention.	INTEF Framework	Questionnaire	Cronbach’s *α*	None	None
Llanes (2023)	2023	Spain	20 pre-service teachers	Mixed method	To explore pre-service teachers’ perceptions of whether their training program and internship enabled them to develop digital teaching competence.	Pre-service teachers felt their training program did not adequately develop their digital teaching competence. More attention is needed to knowledge, skills, and attitudes in education for digital safety.	DigComp Framework	Questionnaire Semi-structured interviews	None	None	None
Çebi et al. (2022)	2022	Turkey	24 pre-service teachers	Mixed method	To analyze pre-service teachers’ progress in digital competence and TPACK after training; to investigate the digital knowledge and skills used in technology integration; to explore correlations between digital competences and TPACK.	Pre-service teachers showed significant improvement in the five areas of DigComp, and the sub-dimensions of design, exertion, ethics, and proficiency after the intervention.	DigComp and TPACK-deep Frameworks (按两个算)	Questionnaire with Likert-type questions Participant diary Focus-group interview	Cronbach’s *α*	None	Data Collection Tools Research Design
Gisbert-Cervera et al. (2022)	2022	Spain	1,350 pre-service teachers	Quantitative	To assess pre-service teachers’ self-perceived and objective TDC levels; to compare TDC scores between trained and untrained groups; to verify the effectiveness of specific TDC training.	Pre-service teachers who received specific digital competence training scored better than those without training.	Frameworks Based on National Standards	Questionnaire Assessment test	None	Degree/grade	Sample Representativeness
Johnson et al. (2024)	2024	Germany	242 pre-service teachers	Mixed method	To evaluate the effectiveness of an extracurricular training program on pre-service teachers’ DigCompEdu competences; to assess changes in their attitudes, competence beliefs, and test-based competences.	Pre-service teachers showed improvement only in confidence using digital technologies for subject-specific purposes after the training program, guiding future curricular refinements.	TPACK Framework	Likert Scale Knowledge tests Interview	Cronbach’s *α*	Teaching strategy	Sample Representativeness Data Collection Tools Research Context/Environment
Romero-Tena et al. (2020)	2020	Spain	535 pre-service early childhood teachers	Quantitative	To investigate pre-service early childhood teachers’ self-perceived digital competence before and after ICT training; to determine whether there are significant differences in self-perception after training; to identify pre-service teachers’ digital competence profiles.	Pre-service teachers’ self-perception of digital competence improved after receiving training, with shifts from lower to higher competence profiles.	Self-Designed Frameworks Based on Literature Reviews	Questionnaire with Likert-type questions	Cronbach’s *α*	Usage frequency	Sample Representativeness
Validation of digital competence tools and frameworks											
Rahimi and Mosalli (2024)	2024	Iran	472 pre-service EFL teachers	Quantitative (PLS-SEM)	To explore the role of pre-service EFL teachers’ 21st-century digital competence in shaping their 21st-century digital skills; to validate the DIGIGLO framework in the Iranian EFL context.	Four areas of digital global competence (DIGIGLO) shaped pre-service EFL teachers’ 21st century digital skills, including critical thinking.	Conceptual Frameworks Developed by Other Authors	Questionnaire	Cronbach’s *α* EFA CFA	None	None
Hairida et al. (2023)	2023	Indonesia	885 pre-service	Quantitative	To validate the adapted digital literacy questionnaire; investigate pre-service chemistry teachers’ digital literacy ability.	The study validated a three-dimensional digital literacy scale for Indonesian preservice chemistry teachers Found them strongest in technical skills, notably weaker in cognitive and social–emotional domains, with no meaningful gender gaps.	Conceptual Frameworks Developed by Other Authors	Questionnaire with Likert-type questions	Cronbach’s *α* Rasch analysis	Gender	Sample Representativeness, Research Design, Research Content/Variables
Quast et al. (2023)	2023	Germany	1386 (prospective) teachers	Quantitative	To develop and validate an instrument to assess professional digital competence beliefs of student teachers, pre-service teachers and teachers; compare group differences.	Seven dimensions of professional digital competence beliefs were identified, with pre-service teachers holding the highest beliefs, followed by in-service teachers.	DigCompEdu Framework	Questionnaire with Likert-type questions	Cronbach’s *α* EFA CFA	Age	Sample Representativeness, Data Collection Tools, Research Context/Environment
Miguel-Revilla et al. (2020)	2020	Spain	50 pre-service teachers	Quantitative	To measure pre-service social studies teachers’ TPACK competence and assess the effectiveness of a project-based learning intervention.	Pre-service teachers had low initial levels of TPACK, but improved significantly after the intervention.	TPACK Framework	Questionnaire with Likert-type questions	Cronbach’s *α*	None	Sample Representativeness Data Collection Tools Research Design
Luik et al. (2018)	2018	Estonia	413 pre-service teachers	Quantitative	To validate a general TPACK measurement instrument in the Estonian context; to describe pre-service teachers’ perceptions of TPACK; to explore relationships between TPACK perceptions and gender, age, and study level.	Pre-service teachers lacked pedagogical knowledge, but perceived they were good at integrating technology into teaching. Differences were found by gender, age, and curricula.	TPACK Framework	Questionnaire with Likert-type questions	Cronbach’s *α* EFA CFA	Gender Age Degree/grade	Sample Representativeness Data Collection Tools
Application of specific digital technologies in teaching											
Kukul (2022)	2022	Turkey	29 pre-service ICT teachers	Mixed method	To evaluate the impact of digital storytelling on pre-service ICT teachers’ perceived TPACK levels; to assess changes in their teaching proficiency self-efficacy; to explore their views on the digital storytelling process.	Digital storytelling significantly improved pre-service ICT teachers’ TPACK and teaching self-efficacy levels.	TPACK Framework	Likert Scale Open-ended interview questions	None	None	Sample Representativeness, Data Collection Tools, Research Design
Belda-Medina and Calvo-Ferrer (2022)	2022	Spain	85 pre-service teachers	Mixed method	To assess pre-service teachers’ digital competence and pedagogical skills for AR integration in CLIL classrooms; to analyze their attitudes toward AR applications; to explore correlations between TPACK and AR attitudes.	Pre-service teachers lacked practical knowledge on AR content creation and implementation, especially in the TPK intersection.	TPACK Framework	Likert Scale Semi-structured focus group discussions	Cronbach’s *α*	None	Sample Representativeness, Research Context/Environment, Data Collection Tools
Polly et al. (2023)	2023	USA	105 pre-service and in-service teachers	Quantitative	To examine pre-service and in-service teachers’ perceptions of digital technologies’ importance, helpfulness, competence, and interest; explore desired supports.	Pre-service teachers rated digital technologies as more important, helpful, and themselves as more competent compared to in-service teachers. Preferences for learning supports also differed.	Self-Designed Frameworks Based on Literature Reviews	Questionnaire with Likert-type questions	Cronbach’s *α*	None	Sample Representativeness Data Collection Tools Research Content/Variables

**Table 3:** Overview of studies grouped by influencing factors.

Specific factors	Study	Year	Country	Sample	Design	Topic	Key findings	Conceptual framework	Instrument	Instrument validation	Influencing factors	Limitations
Studies involving personal background factors												
Gender	Sergeeva et al. (2024)	2024	Russia	163 pre-service teachers	Quantitative	To explore pre-service teachers’ ICT competence beliefs; to identify gender and grade level differences in ICT competence beliefs.	Pre-service teachers had good ICT competence beliefs, with gender differences in a few dimensions and no grade differences. Lower scores were found in analyzing, reflecting, problem-solving, and information/data literacy.	Conceptual Frameworks Developed by Other Authors	Questionnaire with Likert-type questions	Cronbach’s *α*	Gender Degree/grade	Sample Representativeness Data Collection Tools Research Content/Variables
	Çebi and Reisoğlu (2020)	2020	Turkey	518 pre-service teachers	Quantitative	To assess pre-service teachers’ digital competence and explore its variations by gender, branch, and perceived digital competence level.	Pre-service teachers had moderate digital competence, with lower levels in content creation and problem-solving. Gender, branch were factors.	DigComp Framework	Questionnaire with Likert-type questions	None	Gender Discipline branch	Research Content/Variables
	Rodríguez-García et al. (2022)	2022	Spain	698 pre-service teachers	Quantitative	To analyze pre-service teachers’ self-perception of digital competence in communication and collaboration; to explore gender differences in this competence area.	Pre-service teachers had an intermediate level of digital competence for communication and collaboration. Gender differences were found.	DigComp Framework	Likert Scale	Cronbach’s *α*, KMO, Bartlett	Gender	Sample Representativeness, Data Collection Tools
	Torres-Hernández and Gallego-Arrufat (2024)	2024	Spain	1,366 pre-service teachers	Mixed method	To examine pre-service teachers’ self-perceived competences in digital safety; to analyze the differences in digital safety perceptions by gender, age, and degree program.	Pre-service teachers had an intermediate level of digital safety competence. Gender, age, and degree program were differentiating factors in some dimensions. More attention is needed to digital safety education.	Frameworks Based on National Standards	Questionnaire (including dichotomous, open, Likert-type, multiple choice)	Cronbach’s *α* EFA CFA	Gender Age Degree/grade	Data Collection Tools Research Content/Variables
	Palacios-Hidalgo and Huertas-Abril (2022)	2022	Spain	126 pre-service EFL teachers	Quantitative	To examine pre-service EFL teachers’ self-perceived digital literacy; to identify gender and academic year differences in digital competence perceptions; to assess their knowledge of digital competence frameworks.	Pre-service EFL teachers positively self-perceived their digital competence, but felt they did not work enough on it during their university degree. Lower-year students scored higher, and men consistently outperformed women.	Conceptual Frameworks Developed by Other Authors	Questionnaire	Cronbach’s *α*	Gender Degree/grade	Sample Representativeness, Data Collection Tools, Research Content/Variables
	Ata and Yildirim (2019)	2019	Turkey	291 pre-service teachers	Mixed method	To explore pre-service teachers’ perceptions of digital citizenship; to identify differences in digital citizenship perceptions by gender, major, age, parental education level, and internet use; to collect pre-service teachers’ opinions on digital citizenship.	Male pre-service teachers significantly outscored females in digital citizenship, whereas department, high-school type and mother’s education showed no effect Only father’s education mattered. Participants felt proficient in digital communication and participation, credited parents partly, but deemed their major irrelevant.	General Framework	Questionnaire with both open-ended and Likert-type questions	Cronbach’s *α* EFA	Gender Age Discipline branch Usage frequency Parental education level	Sample Representativeness Data Collection Tools Research Content/Variables
	Galindo-Domínguez and Bezanilla (2021)	2021	Spain	200 pre-service teachers	Quantitative	To investigate pre-service teachers’ digital competence profile, and explore differences by degree, gender, course, and age.	Pre-service teachers had a medium level of digital competence, with difficulties in the content creation dimension. Digital competence improved over the years of the degree. No significant differences were found regarding type of university and gender	INTEF Framework	Questionnaire with Likert-type questions	Cronbach’s *α*	Gender Age Degree/Grade	Sample Representativeness Data Collection Tools Research Design Research Context/Environment
	Cañete et al. (2022)	2022	Paraguay	330 pre-service teachers	Quantitative	To assess final-year pre-service teachers’ self-perceived digital competence across four dimensions; to identify differences by gender, age, ICT training and frequency of use.	Preservice teachers had very basic to basic perception about their digital competence. Competence was related to gender, frequency of ICT use, and ICT training. No age differences.	Self-Designed Frameworks Based on Literature Reviews	Likert Scale	Cronbach’s *α*	Gender Age Usage frequency	Sample Representativeness, Data Collection Tools Research Design
	Nurzhanova et al. (2024)	2024	Kazakhstan	209 pre-service teachers	Quantitative	To examine pre-service teachers’ digital literacy and technology use skills; to identify differences by gender and grade level; to explore the predictive role of digital literacy on technology use skills.	Pre-service teachers had high levels of technology use skills and digital literacy, with differences by gender and grade level.	General Framework	Questionnaire with Likert-type questions	Cronbach’s *α*	Gender Degree/grade	None
	Andreasen et al. (2022)	2022	Norway	128 and 118 pre-service teachers	Quantitative	To measure pre-service teachers’ professional digital competence (PDC) before and after teacher education reform; to explore gender differences in ICT self-efficacy; to assess their perception of teacher educators’ PDC needs.	Pre-service teachers showed significant increase in digital competence after teacher education reform. Gender differences were found in ICT self-efficacy.	Self-Designed Frameworks Based on Literature Reviews	Questionnaire	Cronbach’s *α*	Gender National teacher education reform	Data Collection Tools, Research Content/Variables, Research Design
	Yang et al. (2022)	2022	China	250 pre-service, 248 in-service teachers	Quantitative	To measure pre-service and in-service teachers’ perceived digital competence; to explore socio-demographic factors influencing this competence; to compare digital competence between the two teacher groups.	Although ICT awareness is strong, teaching practice remains weak. In-service teachers’ digital competence exceeds that of pre-service peers and correlates significantly with age, teaching years, and educational background.	Frameworks Based on National Standards	Likert Scale	Cronbach’s *α* CFA	Gender Age Degree/grade	Sample Representativeness, Data Collection Tools
	Al-Abdullatif (2019)	2019	Saudi Arabia	113 pre-service teachers	Quantitative	To assess pre-service teachers’ technological knowledge (TK) and TPACK confidence; to identify gender and age differences in TK and TPACK confidence; to evaluate the effectiveness of teacher education programs in fostering TPACK.	Pre-service teachers had very low levels of technological knowledge and TPACK confidence. Female pre-service teachers showed significantly greater confidence and readiness to use ICT for transforming student learning.	TPACK Framework	Questionnaire with Likert-type questions	Cronbach’s *α*	Gender Age	Sample Representativeness
	Xu et al. (2019)	2019	China	905 pre-service teachers	Quantitative	To examine the relationship between pre-service teachers’ interpersonal communication competence (ICC) and digital citizenship; to identify gender differences in ICC and digital citizenship; to explore the predictive power of 10 ICC skills on digital citizenship.	Interpersonal communication competence skills positively predicted digital citizenship among pre-service teachers.	General Framework	Questionnaire with Likert-type questions	Cronbach’s *α*	Gender Communication skills	Sample Representativeness Data Collection Tools Research Content/Variables
	Sergeeva et al. (2023)	2023	Russia	324 pre-service teachers	Quantitative (SEM)	To explore the impact of communication skills (COMP, SELF, SCC, LI-S) on pre-service teachers’ ICT competencies (ICTC-SS, ICTC-ID).	Pre-service teachers’ perceived COMP in communication skills positively influenced their information and communication technology competencies (ICTCs) for student support and instructional design.	General Framework	Likert Scale	Cronbach’s *α*	Gender Discipline branch Communication skills	Sample Representativeness Data Collection Tools Research Content/Variables
	Guillen-Gamez et al. (2020)	2020	Spain	675 pre-service teachers	Quantitative	To evaluate pre-service teachers’ digital competence (attitude, knowledge, use) and compare differences by educational modality (face-to-face vs. blended) and gender.	Pre-service teachers had a medium level of digital competence. No gender differences, but gender differences were found between face-to-face and blended learning modalities.	Self-Designed Frameworks Based on Literature Reviews	Questionnaire with Likert-type questions	Cronbach’s *α*	Gender Education model Attitude	Sample Representativeness Data Collection Tools Research Content/Variables
	Jiménez-Hernández et al. (2020)	2020	Spain	485 pre-service teachers	Quantitative	To measure pre-service secondary school teachers’ digital competence and analyze differences by gender, age (Z-Generation), and branch of knowledge.	Pre-service teachers showed homogeneous digital competence levels, but differences were found in specific areas based on gender, age, and subject area.	DigCompEdu Framework	Questionnaire with Likert-type questions	Cronbach’s *α*	Gender Age Discipline branch	Data Collection Tools
Age	Torres-Hernández and Gallego-Arrufat (2024)	2024	Spain	1,366 pre-service teachers	Mixed method	To examine pre-service teachers’ self-perceived competences in digital safety; to analyze the differences in digital safety perceptions by gender, age, and degree program.	Pre-service teachers had an intermediate level of digital safety competence. Gender, age, and degree program were differentiating factors in some dimensions. More attention is needed to digital safety education.	Frameworks Based on National Standards	Questionnaire (including dichotomous, open, Likert-type, multiple choice)	Cronbach’s *α* EFA CFA	Gender Age Degree/grade	Data Collection Tools Research Content/Variables
	Ata and Yildirim (2019)	2019	Turkey	291 pre-service teachers	Mixed method	To explore pre-service teachers’ perceptions of digital citizenship; to identify differences in digital citizenship perceptions by gender, major, age, parental education level, and internet use; to collect pre-service teachers’ opinions on digital citizenship.	Male pre-service teachers significantly outscored females in digital citizenship, whereas department, high-school type and mother’s education showed no effect Only father’s education mattered. Participants felt proficient in digital communication and participation, credited parents partly, but deemed their major irrelevant.	General Framework	Questionnaire with both open-ended and Likert-type questions	Cronbach’s *α* EFA	Gender Age Discipline branch Usage frequency Parental education level	Sample Representativeness Data Collection Tools Research Content/Variables
	Galindo-Domínguez and Bezanilla (2021)	2021	Spain	200 pre-service teachers	Quantitative	To investigate pre-service teachers’ digital competence profile, and explore differences by degree, gender, course, and age.	Pre-service teachers had a medium level of digital competence, with difficulties in the content creation dimension. Digital competence improved over the years of the degree. No significant differences were found regarding type of university and gender	INTEF Framework	Questionnaire with Likert-type questions	Cronbach’s *α*	Gender Age Degree/Grade	Sample Representativeness Data Collection Tools Research Design Research Context/Environment
	Cañete et al. (2022)	2022	Paraguay	330 pre-service teachers	Quantitative	To assess final-year pre-service teachers’ self-perceived digital competence across four dimensions; to identify differences by gender, age, ICT training and frequency of use.	Preservice teachers had very basic to basic perception about their digital competence. Competence was related to gender, frequency of ICT use, and ICT training. No age differences.	Self-Designed Frameworks Based on Literature Reviews	Likert Scale	Cronbach’s *α*	Gender Age Usage frequency	Sample Representativeness, Data Collection Tools Research Design
	Yang et al. (2022)	2022	China	250 pre-service, 248 in-service teachers	Quantitative	To measure pre-service and in-service teachers’ perceived digital competence; to explore socio-demographic factors influencing this competence; to compare digital competence between the two teacher groups.	Although ICT awareness is strong, teaching practice remains weak. In-service teachers’ digital competence exceeds that of pre-service peers and correlates significantly with age, teaching years, and educational background.	Frameworks Based on National Standards	Likert Scale	Cronbach’s *α* CFA	Gender Age Degree/grade	Sample Representativeness, Data Collection Tools
	Al-Abdullatif (2019)	2019	Saudi Arabia	113 pre-service teachers	Quantitative	To assess pre-service teachers’ technological knowledge (TK) and TPACK confidence; to identify gender and age differences in TK and TPACK confidence; to evaluate the effectiveness of teacher education programs in fostering TPACK.	Pre-service teachers had very low levels of technological knowledge and TPACK confidence. Female pre-service teachers showed significantly greater confidence and readiness to use ICT for transforming student learning.	TPACK Framework	Questionnaire with Likert-type questions	Cronbach’s *α*	Gender Age	Sample Representativeness
	Jiménez-Hernández et al. (2020)	2020	Spain	485 pre-service teachers	Quantitative	To measure pre-service secondary school teachers’ digital competence and analyze differences by gender, age (Z-Generation), and branch of knowledge.	Pre-service teachers showed homogeneous digital competence levels, but differences were found in specific areas based on gender, age, and subject area.	DigCompEdu Framework	Questionnaire with Likert-type questions	Cronbach’s *α*	Gender Age Discipline branch	Data Collection Tools
Attitude	Janeš et al. (2023)	2023	6 countries (Norway, Slovenia, Portugal, Turkey, Ukraine and Jordan)	573 pre-service teachers	Mixed method	To investigate factors influencing pre-service teachers’ future use of digital technologies; assess their attitudes, knowledge, and skills toward DT.	Knowledge, skills, and attitudes toward digital technology were significant predictors of pre-service teachers’ future professional use of digital technology.	Self-Designed Frameworks Based on Literature Reviews	Questionnaire (including open-ended, optional questions and Likert scale)	Cronbach’s *α*	Attitude National/regional differences	Data Collection Tools
	Alnasib (2023)	2023	Saudi Arabia	140 pre-service teachers	Mixed method	To assess pre-service teachers’ self-perceived digital competence; evaluate whether teacher preparation programs qualify them for digital education.	Majority of pre-service teachers rated their digital competence as excellent, but felt their preparation program was only moderately effective in qualifying them for digital education.	DigComp Framework	Questionnaire with both closed-ended and open-ended questions	Cronbach’s *α*	Attitude	Sample Representativeness Data Collection Tools Research Content/Variables
	Guillen-Gamez et al. (2020)	2020	Spain	675 pre-service teachers	Quantitative	To evaluate pre-service teachers’ digital competence (attitude, knowledge, use) and compare differences by educational modality (face-to-face vs. blended) and gender.	Pre-service teachers had a medium level of digital competence. No gender differences, but gender differences were found between face-to-face and blended learning modalities.	Self-Designed Frameworks Based on Literature Reviews	Questionnaire with Likert-type questions	Cronbach’s *α*	Gender Education model Attitude	Sample Representativeness Data Collection Tools Research Content/Variables
Communication skills	Xu et al. (2019)	2019	China	905 pre-service teachers	Quantitative	To examine the relationship between pre-service teachers’ interpersonal communication competence (ICC) and digital citizenship; to identify gender differences in ICC and digital citizenship; to explore the predictive power of 10 ICC skills on digital citizenship.	Interpersonal communication competence skills positively predicted digital citizenship among pre-service teachers.	General Framework	Questionnaire with Likert-type questions	Cronbach’s *α*	Gender Communication skills	Sample Representativeness Data Collection Tools Research Content/Variables
	Sergeeva et al. (2023)	2023	Russia	324 pre-service teachers	Quantitative (SEM)	To explore the impact of communication skills (COMP, SELF, SCC, LI-S) on pre-service teachers’ ICT competencies (ICTC-SS, ICTC-ID).	Pre-service teachers’ perceived COMP in communication skills positively influenced their information and communication technology competencies (ICTCs) for student support and instructional design.	General Framework	Likert Scale	Cronbach’s *α*	Gender Discipline branch Communication skills	Sample Representativeness Data Collection Tools Research Content/Variables
Digital nativity	Yurdakul (2018)	2017	Turkey	1,493 pre-service teachers	Quantitative (SEM)	To explore the relationship between pre-service teachers’ digital nativity and TPACK competencies; to verify whether digital nativity predicts TPACK competencies.	Digital nativity was a significant predictor of pre-service teachers’ TPACK competency.	TPACK-deep Framework	Likert Scale	Cronbach’s *α* EFA CFA	Digital nativity	Sample Representativeness Data Collection Tools Research Content/Variables
Self-efficacy	Lim (2023)	2023	USA	212 pre-service early childhood teachers	Quantitative	To explore the effects of pre-service teachers’ digital literacy and self-efficacy on their perception of AI education for young children.	Pre-service teachers had high critical thinking but low problem-solving in digital literacy. Their digital literacy and self-efficacy positively influenced their perception of AI education for young children.	General Framework	Questionnaire with Likert-type questions	Cronbach’s *α*	Self-efficacy	Sample Representativeness Data Collection Tools
Specific factors	Studies involving educational background and learning experience											
Degree/grade	Sergeeva et al. (2024)	2024	Russia	163 pre-service teachers	Quantitative	To explore pre-service teachers’ ICT competence beliefs; to identify gender and grade level differences in ICT competence beliefs.	Pre-service teachers had good ICT competence beliefs, with gender differences in a few dimensions and no grade differences. Lower scores were found in analyzing, reflecting, problem-solving, and information/data literacy.	Conceptual Frameworks Developed by Other Authors	Questionnaire with Likert-type questions	Cronbach’s *α*	Gender Degree/grade	Sample Representativeness Data Collection Tools Research Content/Variables
	Torres-Hernández and Gallego-Arrufat (2024)	2024	Spain	1,366 pre-service teachers	Mixed method	To examine pre-service teachers’ self-perceived competences in digital safety; to analyze the differences in digital safety perceptions by gender, age, and degree program.	Pre-service teachers had an intermediate level of digital safety competence. Gender, age, and degree program were differentiating factors in some dimensions. More attention is needed to digital safety education.	Frameworks Based on National Standards	Questionnaire (including dichotomous, open, Likert-type, multiple choice)	Cronbach’s *α* EFA CFA	Gender Age Degree/grade	Data Collection Tools Research Content/Variables
	Peled (2021)	2021	Israel	1,265 pre-service teachers	Quantitative	To assess pre-service teachers’ self-perceived digital literacy levels and digital readiness, and explore the predictive effect of background characteristics.	Over 50% of pre-service teachers reported high digital literacy levels. Their sense of readiness for teamwork and ethics was high, but lower in first and advanced order of readiness.	Conceptual Frameworks Developed by Other Authors	Questionnaire with Likert-type questions	Cronbach’s *α*	Degree/Grade	Sample Representativeness Data Collection Tools
	Palacios-Hidalgo and Huertas-Abril (2022)	2022	Spain	126 pre-service EFL teachers	Quantitative	To examine pre-service EFL teachers’ self-perceived digital literacy; to identify gender and academic year differences in digital competence perceptions; to assess their knowledge of digital competence frameworks.	Pre-service EFL teachers positively self-perceived their digital competence, but felt they did not work enough on it during their university degree. Lower-year students scored higher, and men consistently outperformed women.	Conceptual Frameworks Developed by Other Authors	Questionnaire	Cronbach’s *α*	Gender Degree/grade	Sample Representativeness, Data Collection Tools, Research Content/Variables
	Galindo-Domínguez and Bezanilla (2021)	2021	Spain	200 pre-service teachers	Quantitative	To investigate pre-service teachers’ digital competence profile, and explore differences by degree, gender, course, and age.	Pre-service teachers had a medium level of digital competence, with difficulties in the content creation dimension. Digital competence improved over the years of the degree. No significant differences were found regarding type of university and gender	INTEF Framework	Questionnaire with Likert-type questions	Cronbach’s *α*	Gender Age Degree/Grade	Sample Representativeness Data Collection Tools Research Design Research Context/Environment
	Nurzhanova et al. (2024)	2024	Kazakhstan	209 pre-service teachers	Quantitative	To examine pre-service teachers’ digital literacy and technology use skills; to identify differences by gender and grade level; to explore the predictive role of digital literacy on technology use skills.	Pre-service teachers had high levels of technology use skills and digital literacy, with differences by gender and grade level.	General Framework	Questionnaire with Likert-type questions	Cronbach’s *α*	Gender Degree/grade	None
	Yang et al. (2022)	2022	China	250 pre-service, 248 in-service teachers	Quantitative	To measure pre-service and in-service teachers’ perceived digital competence; to explore socio-demographic factors influencing this competence; to compare digital competence between the two teacher groups.	Although ICT awareness is strong, teaching practice remains weak. In-service teachers’ digital competence exceeds that of pre-service peers and correlates significantly with age, teaching years, and educational background.	Frameworks Based on National Standards	Likert Scale	Cronbach’s *α* CFA	Gender Age Degree/grade	Sample Representativeness, Data Collection Tools
Discipline branch	Çebi and Reisoğlu (2020)	2020	Turkey	518 pre-service teachers	Quantitative	To assess pre-service teachers’ digital competence and explore its variations by gender, branch, and perceived digital competence level.	Pre-service teachers had moderate digital competence, with lower levels in content creation and problem-solving. Gender, branch were factors.	DigComp Framework	Questionnaire with Likert-type questions	None	Gender Discipline branch	Research Content/Variables
	Ata and Yildirim (2019)	2019	Turkey	291 pre-service teachers	Mixed method	To explore pre-service teachers’ perceptions of digital citizenship; to identify differences in digital citizenship perceptions by gender, major, age, parental education level, and internet use; to collect pre-service teachers’ opinions on digital citizenship.	Male pre-service teachers significantly outscored females in digital citizenship, whereas department, high-school type and mother’s education showed no effect Only father’s education mattered. Participants felt proficient in digital communication and participation, credited parents partly, but deemed their major irrelevant.	General Framework	Questionnaire with both open-ended and Likert-type questions	Cronbach’s *α* EFA	Gender Age Discipline branch Usage frequency Parental education level	Sample Representativeness Data Collection Tools Research Content/Variables
	Sergeeva et al. (2023)	2023	Russia	324 pre-service teachers	Quantitative (SEM)	To explore the impact of communication skills (COMP, SELF, SCC, LI-S) on pre-service teachers’ ICT competencies (ICTC-SS, ICTC-ID).	Pre-service teachers’ perceived COMP in communication skills positively influenced their information and communication technology competencies (ICTCs) for student support and instructional design.	General Framework	Likert Scale	Cronbach’s *α*	Gender Discipline branch Communication skills	Sample Representativeness Data Collection Tools Research Content/Variables
	Jiménez-Hernández et al. (2020)	2020	Spain	485 pre-service teachers	Quantitative	To measure pre-service secondary school teachers’ digital competence and analyze differences by gender, age (Z-Generation), and branch of knowledge.	Pre-service teachers showed homogeneous digital competence levels, but differences were found in specific areas based on gender, age, and subject area.	DigCompEdu Framework	Questionnaire with Likert-type questions	Cronbach’s *α*	Gender Age Discipline branch	Data Collection Tools
Teaching strategy	Maiier and Koval (2021)	2021	Ukraine	56 pre-service FL teachers	Mixed method	To assess pre-service FL teachers’ readiness to use digital resources, attitudes toward digital competence development, and outline development approaches.	Pre-service foreign-language teachers who used a digital-skills checklist and received regular teacher guidance improved their digital competence significantly more than those who were only consulted by their teachers.	General Framework	Checklists Questionnaire	None	Teaching strategy	Sample Representativeness
	Hirsch and Rubach (2024)	2024	Germany	308 pre-service teachers	Quantitative	To examine the changes in pre-service teachers’ TPK and TPACK over one semester; to evaluate the impact of SQD teaching strategies on the development of TPK and TPACK.	No significant overall change in pre-service teachers’ TPK and TPACK across seminars. The orchestration of SQD teaching strategies positively impacted the development of TPK and TPACK.	TPACK Framework	Likert Scale	Cronbach’s *α* EFA CFA	Teaching strategy	Sample Representativeness Data Collection Tools Research Design Research Content/Variables
National teacher education reform	Andreasen et al. (2022)	2022	Norway	128 and 118 pre-service teachers	Quantitative	To measure pre-service teachers’ professional digital competence (PDC) before and after teacher education reform; to explore gender differences in ICT self-efficacy; to assess their perception of teacher educators’ PDC needs.	Pre-service teachers showed significant increase in digital competence after teacher education reform. Gender differences were found in ICT self-efficacy.	Self-Designed Frameworks Based on Literature Reviews	Questionnaire	Cronbach’s *α*	Gender National teacher education reform	Data Collection Tools, Research Content/Variables, Research Design
Education model	Guillen-Gamez et al. (2020)	2020	Spain	675 pre-service teachers	Quantitative	To evaluate pre-service teachers’ digital competence (attitude, knowledge, use) and compare differences by educational modality (face-to-face vs. blended) and gender.	Pre-service teachers had a medium level of digital competence. No gender differences, but gender differences were found between face-to-face and blended learning modalities.	Self-Designed Frameworks Based on Literature Reviews	Questionnaire with Likert-type questions	Cronbach’s *α*	Gender Education model Attitude	Sample Representativeness Data Collection Tools Research Content/Variables
Specific factors	Studies involving technical use and practice											
Usage frequency	Ata and Yildirim (2019)	2019	Turkey	291 pre-service teachers	Mixed method	To explore pre-service teachers’ perceptions of digital citizenship; to identify differences in digital citizenship perceptions by gender, major, age, parental education level, and internet use; to collect pre-service teachers’ opinions on digital citizenship.	Male pre-service teachers significantly outscored females in digital citizenship, whereas department, high-school type and mother’s education showed no effect Only father’s education mattered. Participants felt proficient in digital communication and participation, credited parents partly, but deemed their major irrelevant.	General Framework	Questionnaire with both open-ended and Likert-type questions	Cronbach’s *α* EFA	Gender Age Discipline branch Usage frequency Parental education level	Sample Representativeness Data Collection Tools Research Content/Variables
	Cañete et al. (2022)	2022	Paraguay	330 pre-service teachers	Quantitative	To assess final-year pre-service teachers’ self-perceived digital competence across four dimensions; to identify differences by gender, age, ICT training and frequency of use.	Preservice teachers had very basic to basic perception about their digital competence. Competence was related to gender, frequency of ICT use, and ICT training. No age differences.	Self-Designed Frameworks Based on Literature Reviews	Likert Scale	Cronbach’s *α*	Gender Age Usage frequency	Sample Representativeness, Data Collection Tools Research Design
Specific Factors	Studies Involving Social and Cultural Factors											
National/regional differences	Janeš et al. (2023)	2023	6 countries (Norway, Slovenia, Portugal, Turkey, Ukraine and Jordan)	573 pre-service teachers	Mixed method	To investigate factors influencing pre-service teachers’ future use of digital technologies; assess their attitudes, knowledge, and skills toward DT.	Knowledge, skills, and attitudes toward digital technology were significant predictors of pre-service teachers’ future professional use of digital technology.	Self-Designed Frameworks Based on Literature Reviews	Questionnaire (including open-ended, optional questions and Likert scale)	Cronbach’s *α*	Attitude National/regional differences	Data Collection Tools
Parental education level	Ata and Yildirim (2019)	2019	Turkey	291 pre-service teachers	Mixed method	To explore pre-service teachers’ perceptions of digital citizenship; to identify differences in digital citizenship perceptions by gender, major, age, parental education level, and internet use; to collect pre-service teachers’ opinions on digital citizenship.	Male pre-service teachers significantly outscored females in digital citizenship, whereas department, high-school type and mother’s education showed no effect Only father’s education mattered. Participants felt proficient in digital communication and participation, credited parents partly, but deemed their major irrelevant.	General Framework	Questionnaire with both open-ended and Likert-type questions	Cronbach’s *α* EFA	Gender Age Discipline branch Usage frequency Parental education level	Sample Representativeness Data Collection Tools Research Content/Variables

**Table 4:** Summary of studies grouped by continents.

Continents	Study	Year	Country	Sample	Design	Topic	Key findings	Conceptual framework	Instrument	Instrument validation	Influencing factors	Limitations
Europe	Hijón-Neira et al. (2023)	2023	Spain	116 pre-service teachers	Quantitative	To test the effect of implementing a digital competence framework in pre-service teacher training; improve their overall digital competence and programming knowledge.	Pre-service teachers significantly improved in digital competence and programming knowledge after the intervention.	INTEF Framework	Questionnaire	Cronbach’s *α*	None	None
	Miguel-Revilla et al. (2020)	2020	Spain	50 pre-service teachers	Quantitative	To measure pre-service social studies teachers’ TPACK competence and assess the effectiveness of a project-based learning intervention.	Pre-service teachers had low initial levels of TPACK, but improved significantly after the intervention.	TPACK	Questionnaire with Likert-type questions	Cronbach’s alpha	None	Sample Representativeness Data Collection Tools Research Design
	Rodríguez-García et al. (2022)	2022	Spain	698 pre-service teachers	Quantitative	To analyze pre-service teachers’ self-perception of digital competence in communication and collaboration; to explore gender differences in this competence area.	Pre-service teachers had an intermediate level of digital competence for communication and collaboration. Gender differences were found.	DigComp Framework	Likert Scale	Cronbach’s *α*, KMO, Bartlett	Gender	Sample Representativeness, Data Collection Tools
	Guillen-Gamez et al. (2020)	2020	Spain	675 pre-service teachers	Quantitative	To evaluate pre-service teachers’ digital competence (attitude, knowledge, use) and compare differences by educational modality (face-to-face vs. blended) and gender.	Pre-service teachers had a medium level of digital competence. No gender differences, but gender differences were found between face-to-face and blended learning modalities.	Self-Designed Frameworks Based on Literature Reviews	Questionnaire with Likert-type questions	Cronbach’s alpha	Gender Education model Attitude	Sample Representativeness Data Collection Tools Research Content/Variables
	Palacios-Hidalgo and Huertas-Abril (2022)	2022	Spain	126 pre-service EFL teachers	Quantitative	To examine pre-service EFL teachers’ self-perceived digital literacy; to identify gender and academic year differences in digital competence perceptions; to assess their knowledge of digital competence frameworks.	Pre-service EFL teachers positively self-perceived their digital competence, but felt they did not work enough on it during their university degree. Lower-year students scored higher, and men consistently outperformed women.	Conceptual Frameworks Developed by Other Authors	Questionnaire	Cronbach’s *α*	Gender Degree/grade	Sample Representativeness, Data Collection Tools, Research Content/Variables
	Galindo-Domínguez and Bezanilla (2021)	2021	Spain	200 pre-service teachers	Quantitative	To investigate pre-service teachers’ digital competence profile, and explore differences by degree, gender, course, and age.	Pre-service teachers had a medium level of digital competence, with difficulties in the content creation dimension. Digital competence improved over the years of the degree. No significant differences were found regarding type of university and gender	INTEF	Questionnaire with Likert-type questions	Cronbach’s *α*	Ggender Age Degree/Grade	Sample Representativeness Data Collection Tools Research Design Research Context/Environment
	Jiménez-Hernández et al. (2020)	2020	Spain	485 pre-service teachers	Quantitative	To measure pre-service secondary school teachers’ digital competence and analyze differences by gender, age (Z-Generation), and branch of knowledge.	Pre-service teachers showed homogeneous digital competence levels, but differences were found in specific areas based on gender, age, and subject area.	DigCompEdu	Questionnaire with Likert-type questions	Cronbach’s alpha	Gender Age Discipline branch	Data Collection Tools
	Belda-Medina and Calvo-Ferrer (2022)	2022	Spain	85 pre-service teachers	Mixed method	To assess pre-service teachers’ digital competence and pedagogical skills for AR integration in CLIL classrooms; to analyze their attitudes toward AR applications; to explore correlations between TPACK and AR attitudes.	Pre-service teachers lacked practical knowledge on AR content creation and implementation, especially in the TPK intersection.	TPACK Framework	Likert Scale Semi-structured focus group discussions	Cronbach’s *α*	None	Sample Representativeness, Research Context/Environment, Data Collection Tools
	Llanes (2023)	2023	Spain	20 pre-service teachers	Mixed method	To explore pre-service teachers’ perceptions of whether their training program and internship enabled them to develop digital teaching competence.	Pre-service teachers felt their training program did not adequately develop their digital teaching competence. More attention is needed to knowledge, skills, and attitudes in education for digital safety.	DigComp	Questionnaire Semi-structured interviews	None	None	None
	Romero-Tena et al. (2020)	2020	Spain	535 pre-service early childhood teachers	Quantitative	To investigate pre-service early childhood teachers’ self-perceived digital competence before and after ICT training; to determine whether there are significant differences in self-perception after training; to identify pre-service teachers’ digital competence profiles.	Pre-service teachers’ self-perception of digital competence improved after receiving training, with shifts from lower to higher competence profiles.	Self-Designed Frameworks Based on Literature Reviews	Questionnaire with Likert-type questions	Cronbach’s alpha	Usage frequency	Sample Representativeness
	Torres-Hernández and Gallego-Arrufat (2024)	2024	Spain	1,366 pre-service teachers	Mixed method	To examine pre-service teachers’ self-perceived competences in digital safety; to analyze the differences in digital safety perceptions by gender, age, and degree program.	Pre-service teachers had an intermediate level of digital safety competence. Gender, age, and degree program were differentiating factors in some dimensions. More attention is needed to digital safety education.	Frameworks Based on National Standards	Questionnaire (including dichotomous, open, Likert-type, multiple choice)	Cronbach’s *α* EFA CFA	Gender Age Degree/grade	Data Collection Tools Research Content/Variables
	Gisbert-Cervera et al. (2022)	2022	Spain	1,350 pre-service teachers	Quantitative	To assess pre-service teachers’ self-perceived and objective TDC levels; to compare TDC scores between trained and untrained groups; to verify the effectiveness of specific TDC training.	Pre-service teachers who received specific digital competence training scored better than those without training.	Frameworks Based on National Standards	Questionnaire Assessment test	None	Degree/grade	Sample Representativeness
	Johnson et al. (2024)	2024	Germany	242 pre-service teachers	Mixed method	To evaluate the effectiveness of an extracurricular training program on pre-service teachers’ DigCompEdu competences; to assess changes in their attitudes, competence beliefs, and test-based competences.	Pre-service teachers showed improvement only in confidence using digital technologies for subject-specific purposes after the training program, guiding future curricular refinements.	TPACK	Likert Scale Knowledge tests Interview	Cronbach’s *α*	Teaching strategy	Sample Representativeness Data Collection Tools Research Context/Environment
	Hirsch and Rubach (2024)	2024	Germany	308 pre-service teachers	Quantitative	To examine the changes in pre-service teachers’ TPK and TPACK over one semester; to evaluate the impact of SQD teaching strategies on the development of TPK and TPACK.	No significant overall change in pre-service teachers’ TPK and TPACK across seminars. The orchestration of SQD teaching strategies positively impacted the development of TPK and TPACK.	TPACK	Likert Scale	Cronbach’s *α* EFA CFA	Teaching strategy	Sample Representativeness Data Collection Tools Research Design Research Content/Variables
	Quast et al. (2023)	2023	Germany	1386 (prospective) teachers	Quantitative	To develop and validate an instrument to assess professional digital competence beliefs of student teachers, pre-service teachers and teachers; compare group differences.	Seven dimensions of professional digital competence beliefs were identified, with pre-service teachers holding the highest beliefs, followed by in-service teachers.	DigCompEdu	Questionnaire with Likert-type questions	Cronbach’s *α* CFA EFA	Age	Sample Representativeness, Data Collection Tools, Research Context/Environment
	Sergeeva et al.	2024	Russia	163 pre-service teachers	Quantitative	To explore pre-service teachers’ ICT competence beliefs; to identify gender and grade level differences in ICT competence beliefs.	Pre-service teachers had good ICT competence beliefs, with gender differences in a few dimensions and no grade differences. Lower scores were found in analyzing, reflecting, problem-solving, and information/data literacy.	Conceptual Frameworks Developed by Other Authors	Questionnaire with Likert-type questions	Cronbach’s *α*	Gender Degree/grade	Sample Representativeness Data Collection Tools Research Content/Variables
	Sergeeva et al. (2023)	2023	Russia	324 pre-service teachers	Quantitative (SEM)	To explore the impact of communication skills (COMP, SELF, SCC, LI-S) on pre-service teachers’ ICT competencies (ICTC-SS, ICTC-ID).	Pre-service teachers’ perceived COMP in communication skills positively influenced their information and communication technology competencies (ICTCs) for student support and instructional design.	General Framework	Likert Scale	Cronbach’s *α*	Gender Discipline branch Communication skills	Sample Representativeness Data Collection Tools Research Content/Variables
	Maiier and Koval (2021)	2021	Ukraine	56 pre-service FL teachers	Mixed method	To assess pre-service FL teachers’ readiness to use digital resources, attitudes toward digital competence development, and outline development approaches.	Pre-service foreign-language teachers who used a digital-skills checklist and received regular teacher guidance improved their digital competence significantly more than those who were only consulted by their teachers.	General Framework	Checklists Questionnaire	None	Teaching strategy	Sample Representativeness
	Andreasen et al. (2022)	2022	Norway	128 and 118 pre-service teachers	Quantitative	To measure pre-service teachers’ professional digital competence (PDC) before and after teacher education reform; to explore gender differences in ICT self-efficacy; to assess their perception of teacher educators’ PDC needs.	Pre-service teachers showed significant increase in digital competence after teacher education reform. Gender differenceswere found in ICT self-efficacy.	Self-Designed Frameworks Based on Literature Reviews	Questionnaire	Cronbach’s *α*	Gender National teacher education reform	Data Collection Tools, Research Content/Variables, Research Design
	McGarr and McDonagh (2021)	2021	Ireland	208 pre-service teachers	Mixed method	To explore pre-service teachers’ digital competence on entry into initial teacher education, including technical skills, cyber ethics knowledge, and attitudes toward technology in teaching.	Pre-service teachers were active technology users, but reported lower skills in using other digital technologies. Their knowledge of cyber ethics and associated practices also varied.	Conceptual Frameworks Developed by Other Authors	Questionnaire with both open-ended and Likert-type questions	None	None	Data Collection Tools Research Content/Variables
	Luik et al. (2018)	2018	Estonia	413 pre-service teachers	Quantitative	To validate a general TPACK measurement instrument in the Estonian context; to describe pre-service teachers’ perceptions of TPACK; to explore relationships between TPACK perceptions and gender, age, and study level.	Pre-service teachers lacked pedagogical knowledge, but perceived they were good at integrating technology into teaching. Differences were found by gender, age, and curricula.	TPACK	Questionnaire with Likert-type questions	Cronbach’s *α* EFA CFA	Gender Age Degree/grade	Sample Representativeness Data Collection Tools
	Janeš et al. (2023)	2023	6 countries (Norway, Slovenia, Portugal, Turkey, Ukraine and Jordan)	573 pre-service teachers	Mixed method	To investigate factors influencing pre-service teachers’ future use of digital technologies; assess their attitudes, knowledge, and skills toward DT.	Knowledge, skills, and attitudes toward digital technology were significant predictors of pre-service teachers’ future professional use of digital technology.	Self-Designed Frameworks Based on Literature Reviews	Questionnaire (including open-ended, optional questions and Likert scale)	Cronbach’s *α*	Attitude National/regional differences	Data Collection Tools
Asia	Çebi et al. (2022)	2022	Turkey	24 pre-service teachers	Mixed method	To analyze pre-service teachers’ progress in digital competence and TPACK after training; to investigate the digital knowledge and skills used in technology integration; to explore correlations between digital competences and TPACK.	Pre-service teachers showed significant improvement in the five areas of DigComp, and the sub-dimensions of design, exertion, ethics, and proficiency after the intervention.	DigComp and TPACK-deep	Questionnaire with Likert-type questions Participant diary Focus-group interview	Cronbach’s *α*	None	Data Collection Tools Research Design
	Çebi and Reisoğlu (2020)	2020	Turkey	518 pre-service teachers	Quantitative	To assess pre-service teachers’ digital competence and explore its variations by gender, branch, and perceived digital competence level.	Pre-service teachers had moderate digital competence, with lower levels in content creation and problem-solving. Gender, branch were factors.	DigComp	Questionnaire with Likert-type questions	None	Gender Discipline branch	Research Content/Variables
	Kukul (2022)	2022	Turkey	29 pre-service ICT teachers	Mixed method	To evaluate the impact of digital storytelling on pre-service ICT teachers’ perceived TPACK levels; to assess changes in their teaching proficiency self-efficacy; to explore their views on the digital storytelling process.	Digital storytelling significantly improved pre-service ICT teachers’ TPACK and teaching self-efficacy levels.	TPACK Framework	Likert Scale Open-ended interview questions	None	None	Sample Representativeness, Data Collection Tools, Research Design
	Yurdakul	2017	Turkey	1,493 pre-service teachers	Quantitative, SEM	To explore the relationship between pre-service teachers’ digital nativity and TPACK competencies; to verify whether digital nativity predicts TPACK competencies.	Digital nativity was a significant predictor of pre-service teachers’ TPACK competency.	TPACK-deep	Likert Scale	Cronbach’s *α* EFA CFA	Digital nativity	Sample Representativeness Data Collection Tools Research Content/Variables
	Ata and Yildirim (2019)	2019	Turkey	291 pre-service teachers	Mixed method	To explore pre-service teachers’ perceptions of digital citizenship; to identify differences in digital citizenship perceptions by gender, major, age, parental education level, and internet use; to collect pre-service teachers’ opinions on digital citizenship.	Male pre-service teachers significantly outscored females in digital citizenship, whereas department, high-school type and mother’s education showed no effect Only father’s education mattered. Participants felt proficient in digital communication and participation, credited parents partly, but deemed their major irrelevant.	General Framework	Questionnaire with both open-ended and Likert-type questions	Cronbach’s *α* EFA	Gender Age Discipline branch Usage frequency Parental education level	Sample Representativeness Data Collection Tools Research Content/Variables
	Al-Abdullatif (2019)	2019	Saudi Arabia	113 pre-service teachers	Quantitative	To assess pre-service teachers’ technological knowledge (TK) and TPACK confidence; to identify gender and age differences in TK and TPACK confidence; to evaluate the effectiveness of teacher education programs in fostering TPACK.	Pre-service teachers had very low levels of technological knowledge and TPACK confidence. Female pre-service teachers showed significantly greater confidence and readiness to use ICT for transforming student learning.	TPACK	Questionnaire with Likert-type questions	Cronbach’s *α*	Gender Age	Sample Representativeness
	Alnasib	2023	Saudi Arabia	140 pre-service teachers	Mixed method	To assess pre-service teachers’ self-perceived digital competence; evaluate whether teacher preparation programs qualify them for digital education.	Majority of pre-service teachers rated their digital competence as excellent, but felt their preparation program was only moderately effective in qualifying them for digital education.	DigComp	Questionnaire with both colsed-ended and open-ended questions	Cronbach’s *α*	Attitude	Sample Representativeness Data Collection Tools Research Content/Variables
	Yang et al. (2022)	2022	China	250 pre-service, 248 in-service teachers	Quantitative	To measure pre-service and in-service teachers’ perceived digital competence; to explore socio-demographic factors influencing this competence; to compare digital competence between the two teacher groups.	Although ICT awareness is strong, teaching practice remains weak. In-service teachers’ digital competence exceeds that of pre-service peers and correlates significantly with age, teaching years, and educational background.	Frameworks Based on National Standards	Likert Scale	Cronbach’s *α* CFA	Gender Age Degree/grade	Sample Representativeness, Data Collection Tools
	Xu et al. (2019)	2019	China	905 pre-service teachers	Quantitative	To examine the relationship between pre-service teachers’ interpersonal communication competence (ICC) and digital citizenship; to identify gender differences in ICC and digital citizenship; to explore the predictive power of 10 ICC skills on digital citizenship.	Interpersonal communication competence skills positively predicted digital citizenship among pre-service teachers.	General Framework	Questionnaire with Likert-type questions	Cronbach’s *α*	Gender Communication skills	Sample Representativeness Data Collection Tools Research Content/Variables
	Hairida et al.	2023	Indonesia	885 pre-service	Quantitative	To validate the adapted digital literacy questionnaire; investigate pre-service chemistry teachers’ digital literacy ability.	The study validated a threedimensional digitalliteracy scale for Indonesian preservice chemistry teachers Found them strongest in technical skills, notably weaker in cognitive and social–emotional domains, with no meaningful gender gaps.	Conceptual Frameworks Developed by Other Authors	Questionnaire with Likert-type questions	Cronbach’s *α* Rasch analysis	Gender	Sample Representativeness, Research Design, Research Content/Variables
	Nurzhanova et al. (2024)	2024	Kazakhstan	209 pre-service teachers	Quantitative	To examine pre-service teachers’ digital literacy and technology use skills; to identify differences by gender and grade level; to explore the predictive role of digital literacy on technology use skills.	Pre-service teachers had high levels of technology use skills and digital literacy, with differences by gender and grade level.	General Framework	Questionnaire with Likert-type questions	Cronbach’s *α*	Gender Degree/grade	None
	Janeš et al. (2023)	2023	6 countries (Norway, Slovenia, Portugal, Turkey, Ukraine and Jordan)	573 pre-service teachers	Mixed method	To investigate factors influencing pre-service teachers’ future use of digital technologies; assess their attitudes, knowledge, and skills toward DT.	Knowledge, skills, and attitudes toward digital technology were significant predictors of pre-service teachers’ future professional use of digital technology.	Self-Designed Frameworks Based on Literature Reviews	Questionnaire (including open-ended, optional questions and Likert scale)	Cronbach’s *α*	Attitude National/regional differences	Data Collection Tools
	Peled (2021)	2021	Israel	1,265 pre-service teachers	Quantitative	To assess pre-service teachers’ self-perceived digital literacy levels and digital readiness, and explore the predictive effect of background characteristics.	Over 50% of pre-service teachers reported high digital literacy levels. Their sense of readiness for teamwork and ethics was high, but lower in first and advanced order of readiness.	Conceptual Frameworks Developed by Other Authors	Questionnaire with Likert-type questions	Cronbach’s *α*	Degree/Grade	Sample Representativeness Data Collection Tools
	Rahimi and Mosalli (2024)	2024	Iran	472 pre-service EFL teachers	Quantitative (PLS-SEM)	To explore the role of pre-service EFL teachers’ 21st-century digital competence in shaping their 21st-century digital skills; to validate the DIGIGLO framework in the Iranian EFL context.	Four areas of digital global competence (DIGIGLO) shaped pre-service EFL teachers’ 21st century digital skills, including critical thinking.	Conceptual Frameworks Developed by Other Authors	Questionnaire	Cronbach’s *α* EFA CFA	None	None
Americas	Cañete et al. (2022)	2022	Paraguay	330 pre-service teachers	Quantitative	To assess final-year pre-service teachers’ self-perceived digital competence across four dimensions; to identify differences by gender, age, ICT training and frequency of use.	Preservice teachers had very basic to basic perception about their digital competence. Competence was related to gender, frequency of ICT use, and ICT training. No age differences.	Self-Designed Frameworks Based on Literature Reviews	Likert Scale	Cronbach’s *α*	Gender Age Usage frequency	Sample Representativeness, Data Collection Tools Research Design
	Polly et al. (2023)	2023	USA	105 pre-service and in-service teachers	Quantitative	To examine pre-service and in-service teachers’ perceptions of digital technologies’ importance, helpfulness, competence, and interest; explore desired supports.	Pre-service teachers rated digital technologies as more important, helpful, and themselves as more competent compared to in-service teachers. Preferences for learning supports also differed.	Self-Designed Frameworks Based on Literature Reviews	Questionnaire with Likert-type questions	Cronbach’s *α*	None	Sample Representativeness Data Collection Tools Research Content/Variables
	Lim (2023)	2023	USA	212 pre-service early childhood teachers	Quantitative	To explore the effects of pre-service teachers’ digital literacy and self-efficacy on their perception of AI education for young children.	Pre-service teachers had high critical thinking but low problem-solving in digital literacy. Their digital literacy and self-efficacy positively influenced their perception of AI education for young children.	General Framework	Questionnaire with Likert-type questions	Cronbach’s *α*	Self-efficacy	Sample Representativeness Data Collection Tools

### 4.1 Trends in published articles over time

**Figure 2:**
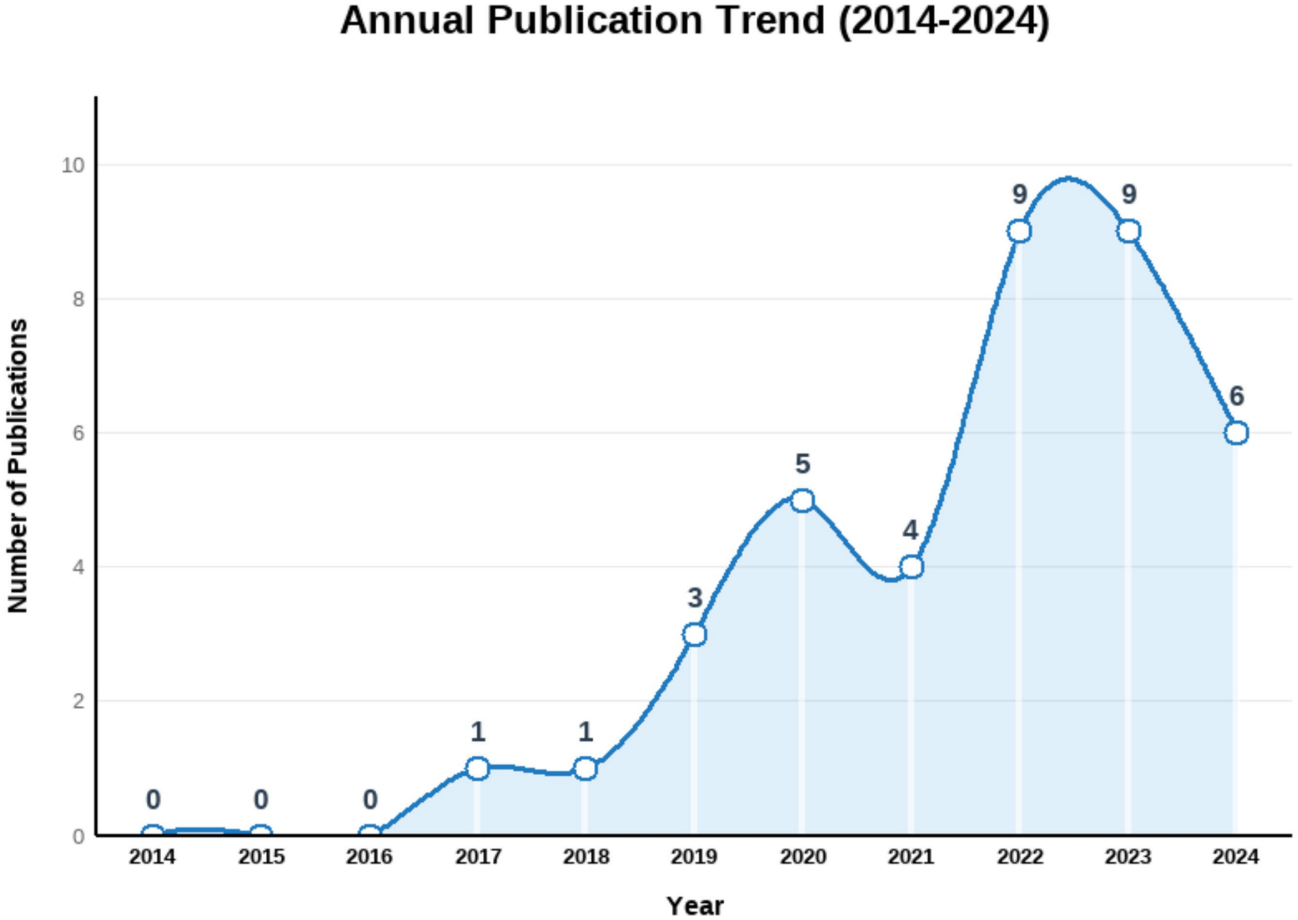
Publication volume trends from 2014 to 2024.

A temporal analysis of the published articles reveals a notable increase in publications on digital teaching competence from 2014 to 2024. It is worth highlighting that research focusing on assessing pre-service teachers’ digital competence began to emerge in 2017, followed by a steady annual growth. Specifically, three articles were published in 2019, and this number rose to five in 2020. Between 2021 and 2023, the number of publications surged significantly, reaching a peak during this period. Although the number of articles declined slightly in 2024, it remained at a relatively high level. Overall, the trend shows an initial period of minimal research, followed by rapid growth, stabilization, and a slight decline as illustrated in Figure 2.

### 4.2 Digital competence frameworks for pre-service teachers

**Figure 3:**
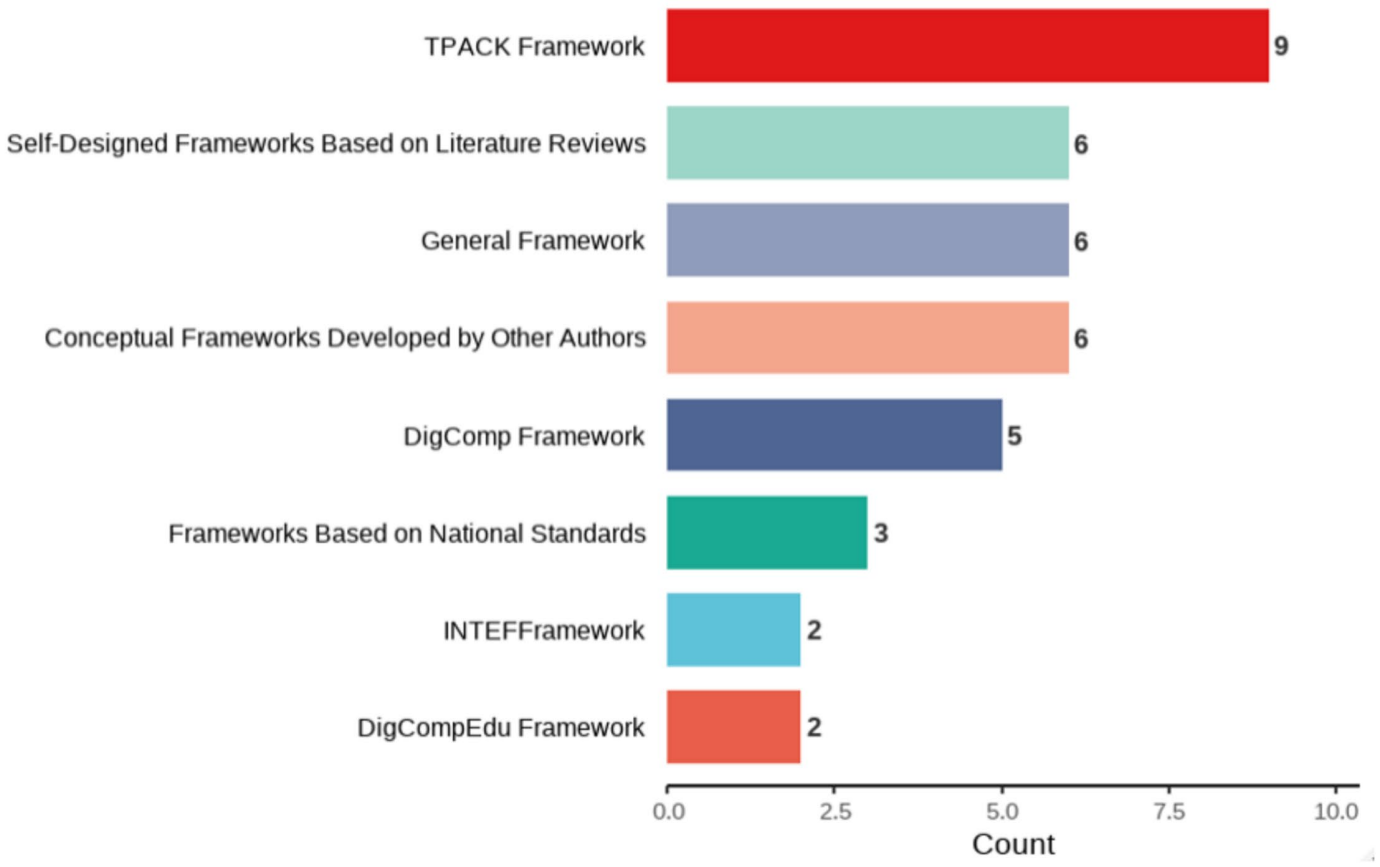
Digital competence frameworks in the included studies.

As shown in Figure 3, the conceptual frameworks used to define pre-service teachers’ digital competence in the selected studies exhibit significant diversity. Researchers have adopted various frameworks based on different theoretical perspectives and practical needs to explore teachers’ digital competence.

According to the analysis, six articles employed general concepts, viewing digital competence as part of a broader professional or civic competence framework. Among these articles, one study applied the concept of digital citizenship based on national standards (e.g., ISTE standards), emphasizing teachers’ civic responsibilities and competencies in a digital society. The remaining 32 articles utilized specific digital competence frameworks.

Nine articles adopted the TPACK framework, which emphasizes the importance of technological, pedagogical, and content knowledge, as well as their integration. Two of these studies employed the TPACK-deep framework (Çebi et al., 2022; Yurdakul, 2018), which further defines and extends TPACK. Additionally, Çebi et al. (2022) incorporated both the TPACK framework and the DigComp framework in their study.

Furthermore, the DigComp framework, originally developed to define digital competence for the general population, and its education-specific version, DigCompEdu, specifically tailored for educators, also garnered attention in other studies, with five and two articles adopting these frameworks, respectively. The increasing use of these frameworks suggests a move toward more consistent approaches to defining and assessing digital competence in teacher education. The INTEF framework was also adopted in two articles, providing a European perspective on teachers’ digital competence.

Additionally, six articles employed conceptual frameworks developed by other scholars from their respective studies, characterized by theoretical perspectives and practical applications. For example, Peled (2021) based their study on the Seven Domains of Digital Literacy (SDDL) model, developed and validated by Kurtz and Peled (2016), which identifies levels of digital readiness and competence.

Notably, six articles developed self-designed conceptual frameworks based on literature reviews, aiming to synthesize the strengths of existing research to provide a more comprehensive definition of teachers’ digital competence. For instance, Andreasen et al. (2022) developed the theoretical concept of professional digital competence (PDC) after reviewing the relevant literature; Polly et al. (2023) adapted the Technology Integration Readiness Framework to propose their own conceptualization and dimensions of digital competence; and Romero-Tena et al. (2020) proposed six dimensions of digital competence, including Technological Literacy, based on various institutional models or frameworks. These cases highlight efforts to refine existing models and capture the diverse aspects of teachers’ digital competence.

Moreover, three articles employed frameworks based on national standards, although these frameworks were not widely used. These frameworks typically reflect the educational policies and practical needs of specific countries. For example, Torres-Hernández and Gallego-Arrufat (2024) referenced Spain’s Digital Safety Education Reference Framework (MCDD) to examine pre-service teachers’ digital safety competence; Yang et al. (2022) explored digital competence using a conceptual framework validated in the Chinese context; and Gisbert-Cervera et al. (2022) referenced the COMDID framework established by the Catalan government. These nationally grounded frameworks provide valuable context-specific insights.

### 4.3 Research themes and findings

**Figure 4:**
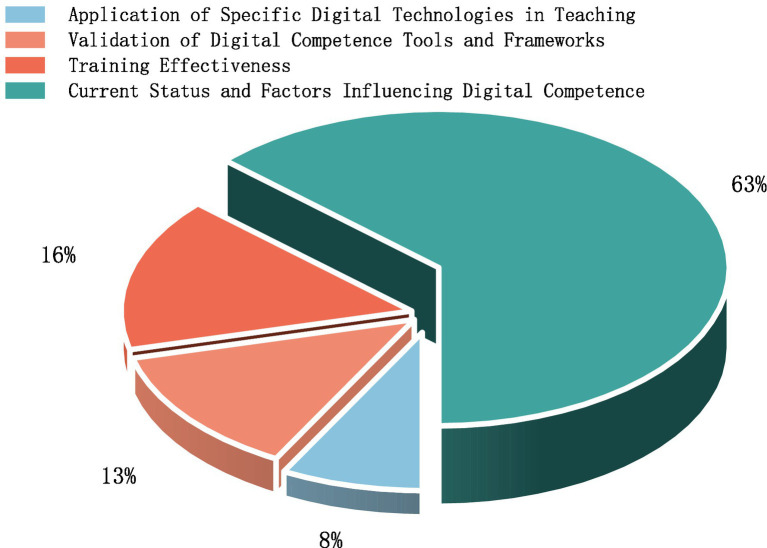
Research themes of the studies.

Figure 4 illustrates the research themes of all selected studies, which we categorized into four groups, as follows: (1) a significant proportion of studies (*n* = 24, approximately 63%) focused on the current status of digital competence among pre- or in-service teachers and its influencing factors; (2) six studies (approximately 16%) investigated the effectiveness of training programs; (3) five studies (approximately 13%) concentrated on the validation of digital competence frameworks and tools, and (4) three studies (approximately 8%) specifically examined the application of particular digital technologies (detailed information provided in Table 2).

#### 4.3.1 Current status and factors influencing digital competence

Most of the selected studies (*n* = 24) explored the current status of pre-service teachers’ digital competence levels and attempted to explain the variations across dimensions through factors such as gender, age, academic discipline, and educational background. Among these, seven articles focused more on investigating the influencing factors than evaluating digital competence itself.

The current status of pre-service teachers’ digital competence has been assessed from multiple perspectives in these studies, including their self-perception of digital technologies, levels of digital literacy, and performance across various domains. Regarding overall digital competence levels, five articles reported that pre-service teachers exhibited high or satisfactory levels of digital literacy and technology use skills or held highly positive self-perceptions. Conversely, three articles indicated that pre-service early childhood and primary school teachers demonstrated average performance across all digital competence dimensions.

In terms of specific dimensions, despite variations in measurement frameworks, multiple studies highlighted that pre-service teachers exhibited lower proficiency in digital content creation, problem-solving, and technology integration practices, while demonstrating higher competence in communication and collaboration. However, some studies reported significant variations within the same dimensions. For instance, Cañete et al. (2022) found that future teachers in Paraguay exhibited high competence in “technical” and “ethical and legal aspects,” whereas McGarr and McDonagh (2021) revealed that Irish pre-service teachers had limited understanding and mastery of cyber ethics.

**Figure 5:**
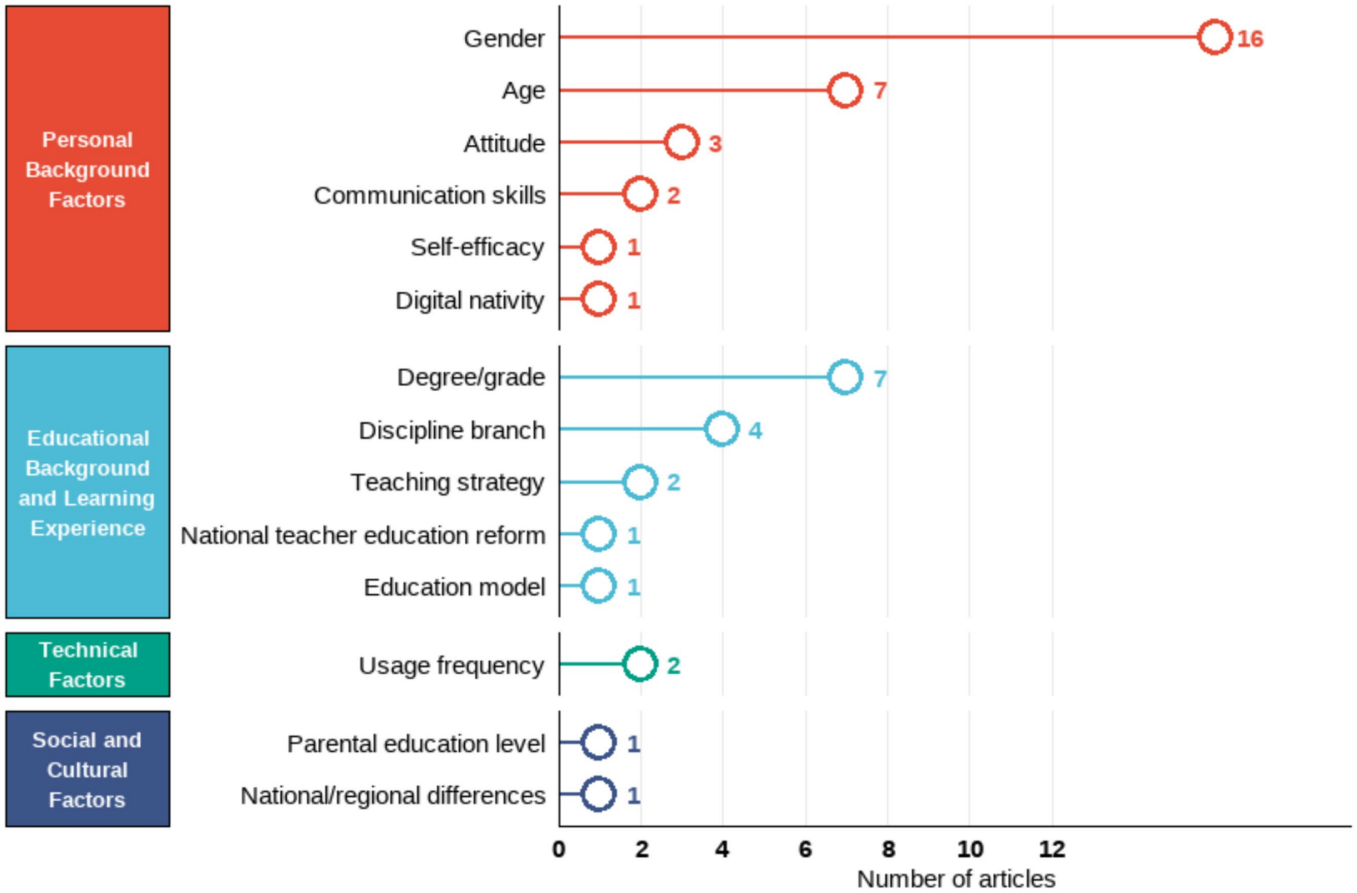
Influencing factors and number of articles.

The influencing factors identified in these studies fall into four categories, as illustrated in Figure 5, with detailed information provided in Table 3: personal background factors (e.g., gender and age), educational background and learning experiences (e.g., degree level and academic year), technology use and practice (e.g., frequency of use), and social and cultural factors (e.g., national or regional differences). A range of factors within these categories was found to affect pre-service teachers’ digital competence. Gender and age were the most frequently examined personal background factors, addressed in 16 and 7 studies, respectively, indicating their widespread recognition as significant influences on digital competence. In terms of educational background and learning experiences, degree/grade and discipline branch (affiliated department) were the main focuses. Social and cultural factors, including national/regional differences and parental education level, were each examined in 1 study, indicating these areas remain underexplored. Technology use and practice, particularly frequency of use, were explored in only two studies, suggesting that this factor has not yet been thoroughly validated. Factors such as self-efficacy, digital nativity, and teaching strategies (e.g., the SQD model) were less frequently studied but show significant potential in influencing digital competence and merit further research. In the following sections, we will discuss the findings for each category in detail.

Regarding personal background factors, some studies suggest that males tend to perceive their digital competence more positively than females. For example, males outperformed females in information and data literacy, digital content creation, security, and problem-solving, while females demonstrated stronger communication and collaboration skills (Jiménez-Hernández et al., 2020; Çebi and Reisoğlu, 2020). However, Al-Abdullatif (2019) found that female pre-service teachers exhibited greater confidence and willingness to engage in ICT practices to enhance student learning outcomes. Although some studies reported no significant gender differences (Sergeeva et al., 2024; Guillen-Gamez et al., 2020), gender remains a critical variable influencing digital competence.

Additionally, multiple studies indicated a positive correlation between age and certain dimensions of digital competence (Yang et al., 2022; Galindo-Domínguez and Bezanilla, 2021). Older pre-service teachers generally demonstrated higher levels of digital competence, while those born after 1990, having grown up in a digitalized environment, scored higher across various domains (Jiménez-Hernández et al., 2020). Studies on digital nativity also revealed that pre-service teachers with higher levels of digital nativity exhibited stronger TPACK (Technological Pedagogical Content Knowledge) competencies (Yurdakul, 2018). Furthermore, self-efficacy (Lim, 2023) and attitudes (Alnasib, 2023; Sergeeva et al., 2024) were found to correlate significantly with digital competence. Specifically, pre-service teachers with positive attitudes toward digital technologies and higher self-efficacy demonstrated enhanced digital competence. Communication skills were also identified as crucial, particularly in digital collaboration and teamwork, where strong communication abilities significantly improved pre-service teachers’ performance in technology-supported instructional design (Xu et al., 2019; Sergeeva et al., 2023).

Regarding educational background and learning experiences, significant differences were observed among pre-service teachers across different academic disciplines, with students in computer education or instructional technology outperforming peers in all competence domains, in all areas of digital competence, likely due to greater exposure to digital technologies in their curricula (Çebi and Reisoğlu, 2020). Additionally, degree level and academic year were examined in seven studies, yet the findings remain inconclusive. Peled (2021) found that graduate students excelled in teamwork and ethical preparedness, and digital competence rose across grades. Galindo-Domínguez and Bezanilla (2021), suggesting that digital competence improves with higher educational attainment. However, Palacios-Hidalgo and Huertas-Abril (2022) reported lower-year students scored higher in some areas and Sergeeva et al. (2024) found no significant relationship between degree and digital competence.

Research on teaching strategies, especially the SQD model revealed that pre-service teachers exposed to SQD-aligned strategies demonstrated more significant development in TPK (Technological Pedagogical Knowledge) and TPACK competencies, underscoring the importance of teaching strategies in fostering digital competence (Hirsch and Rubach, 2024). Andreasen et al. (2022) examined national teacher education reforms, finding that post-reform pre-service teachers demonstrated significantly enhanced classroom technology integration abilities, highlighting the impact of policy environments on digital competence development.

Technology use and practice were found to influence pre-service teachers’ digital competence, although evidence is mixed. Cañete et al. (2022) reported a positive correlation between the frequency of information and communication technology use and various dimensions of digital competence, indicating that pre-service teachers who used the internet more frequently demonstrated superior digital competence. However, Ata and Yildirim (2019) found no significant relationship between digital citizenship levels and internet use frequency.

Finally, social and cultural factors were found to play a significant role in shaping pre-service teachers’ digital competence. Janeš et al. (2023) identified notable differences in pre-service teachers’ familiarity with and confidence in using digital tools across different countries, emphasizing the influence of cultural and social environments on digital competence development. Additionally, paternal education level was found to correlate with pre-service teachers’ digital citizenship levels, suggesting that family background influences digital competence development (Ata and Yildirim, 2019). Furthermore, one study explored the impact of digital literacy on other factors, revealing that digital literacy positively influenced technology acceptance. Lim (2023) found that pre-service teachers’ digital literacy and self-efficacy were positively correlated with their perceptions of AI education for young children.

#### 4.3.2 Training effectiveness

As shown in Figure 4, six articles (16%) explored the impact of different training programs on the development of pre-service teachers’ digital competence, aiming to provide empirical evidence to inform teacher training. Specifically, these studies approached the topic from various perspectives, collectively examining the training needs and outcomes of pre-service teachers in terms of digital competence, knowledge, skills, and related teaching abilities. Several studies applied established frameworks, such as the DigComp framework (Çebi et al., 2022) and the INTEF framework (Hijón-Neira et al., 2023), to examine how training programs influence pre-service teachers’ competence and knowledge. Similarly, Romero-Tena et al. (2020) and Johnson et al. (2024) used pre- and post-tests to explore changes in pre-service teachers’ attitudes, self-efficacy beliefs, or self-perceptions regarding technology, revealing positive shifts in their self-perceived digital skills after training. Additionally, Gisbert-Cervera et al. (2022) and Llanes (2023) conducted region-specific studies in Catalonia, examining whether pre-service teachers required targeted training, as well as their perceptions of whether such training could enhance their digital competence.

Multiple studies affirmed the positive impact of training on pre-service teachers’ digital competence, particularly under specific frameworks or programs where significant improvements in overall digital competence and related teaching abilities were observed (Çebi et al., 2022; Romero-Tena et al., 2020; Hijón-Neira et al., 2023). However, the effectiveness of current programs remains limited in some areas. For instance, some training programs showed minimal impact on improving pre-service teachers’ attitudes, self-efficacy beliefs, and perceptions of digital technologies and learning (Johnson et al., 2024). This may be attributed to shortcomings in current training programs, particularly in terms of technology integration and innovative application. For example, Llanes (2023) highlighted the lack of courses focused on technology integration, with pre-service teachers demonstrating notable deficiencies in areas such as civic engagement, digital identity, netiquette, programming, copyright, licensing, security, and the innovative and creative use of digital technologies. These findings suggest that pre-service teachers require continuous, comprehensive, and tailored training to enhance their digital competence across all dimensions, as current programs are inconsistent and do not fully meet the demands of the education system (Gisbert-Cervera et al., 2022).

#### 4.3.3 Validation of digital competence tools and frameworks

Five articles (13%) were dedicated to developing and validating tools or frameworks for assessing pre-service teachers’ digital competence, ensuring their reliability and effectiveness. For example, Rahimi and Mosalli (2024) and Hairida et al. (2023) validated digital competence frameworks or assessment questionnaires in specific contexts. The former validated the DIGIGLO framework in the context of Iranian EFL and language education, while the latter validated a digital literacy questionnaire in the Indonesian context. Additionally, Miguel-Revilla et al. (2020) and Luik et al. (2018) developed or tested assessment tools based on the TPACK model, whereas Quast et al. (2023) developed and validated a tool based on the DigCompEdu framework to assess pre-service teachers’ professional digital competence beliefs. By validating frameworks and tools across diverse contexts, these studies contribute to standardizing measurement approaches, providing foundations for future research and teacher training initiatives.

#### 4.3.4 Application of specific digital technologies in teaching

Three articles (8%) focused on the effectiveness of specific digital technologies (e.g., augmented reality and digital storytelling) in teaching, as well as pre-service teachers’ acceptance and proficiency in using these technologies. For example, Kukul (2022) found that digital storytelling significantly positively influenced pre-service teachers’ TPACK skills and teaching self-efficacy. Additionally, pre-service teachers held positive attitudes toward the use of augmented reality in language teaching, though they faced challenges in integrating that technology (Belda-Medina and Calvo-Ferrer, 2022). However, Belda-Medina and Calvo-Ferrer (2022) noted that pre-service teachers face challenges in integrating digital methods with emerging technologies highlighting the need for future research and practice to develop strategies that better support the effective use of emerging technologies in teacher education.

**Figure 6:**
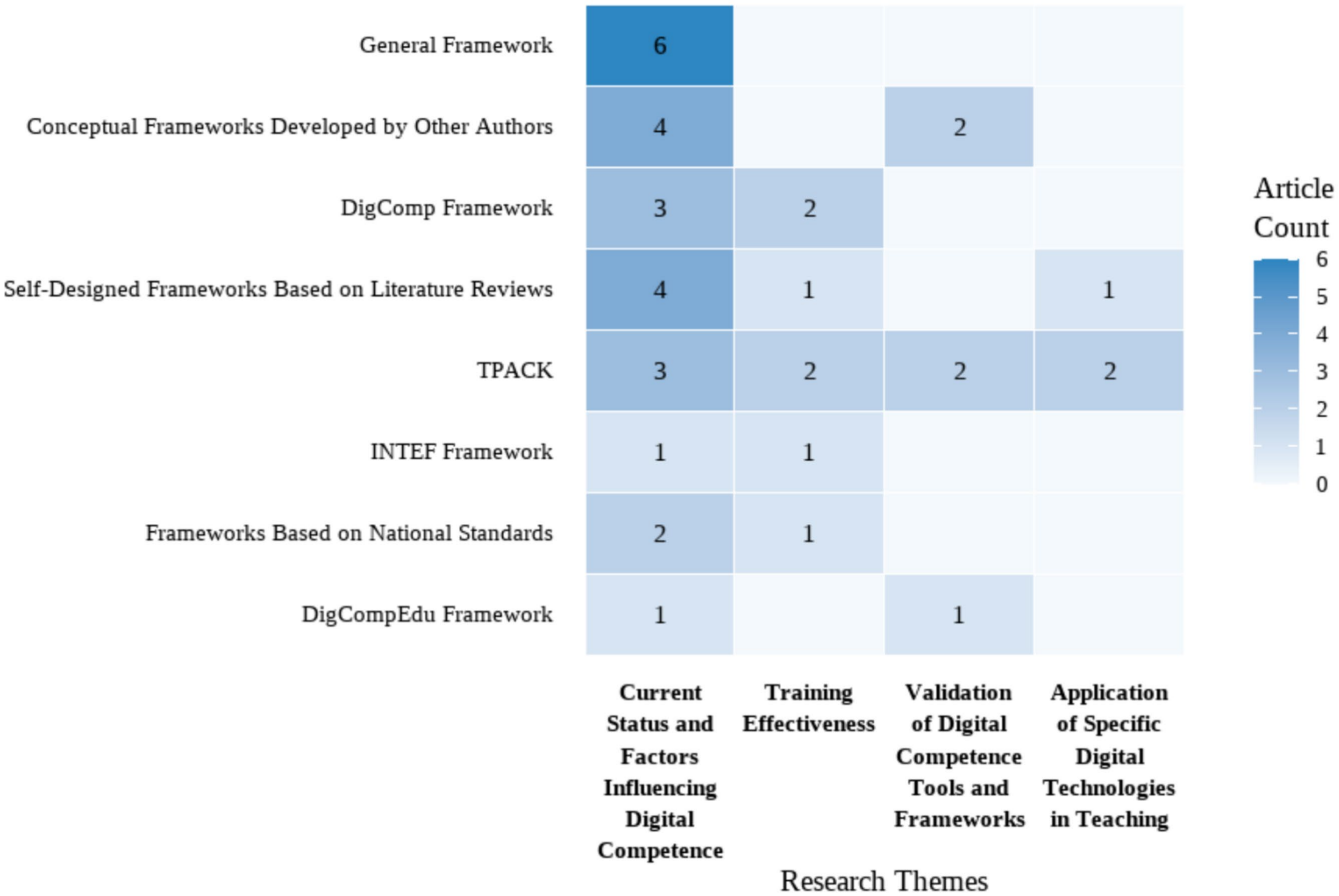
Framework application distribution across research themes.

To illustrate how these varied frameworks were applied across the four main research agendas, Figure 6 uses color intensity to show the number of studies associated with each framework–theme combination.

Among the four research themes, the “Current Status and Factors Influencing Digital Competence” theme saw the most concentrated framework applications: the General Framework (6 studies) dominated here, followed by Conceptual Frameworks Developed by Other Authors (4) and Self-Designed Frameworks Based on Literature Reviews (4). For the “Training Effectiveness” theme, frameworks were more evenly distributed, with DigComp Framework (2) and TPACK (2) being the most frequently used. The “Validation of Digital Competence Tools and Frameworks” and “Application of Specific Digital Technologies in Teaching” themes had relatively sparse framework coverage: only TPACK (2 studies each) and a small number of other frameworks (e.g., DigCompEdu Framework, 1 study) were applied in these two themes.

### 4.4 Research methods of the selected studies

We investigated the research regions, data collection methods, and tools. In analyzing current research methods in the field of education, this study comprehensively examined several key characteristics, including the research regions, types of methods, data collection tools, and tool validation approaches, to reveal their diversity and geographical distribution.

**Figure 7:**
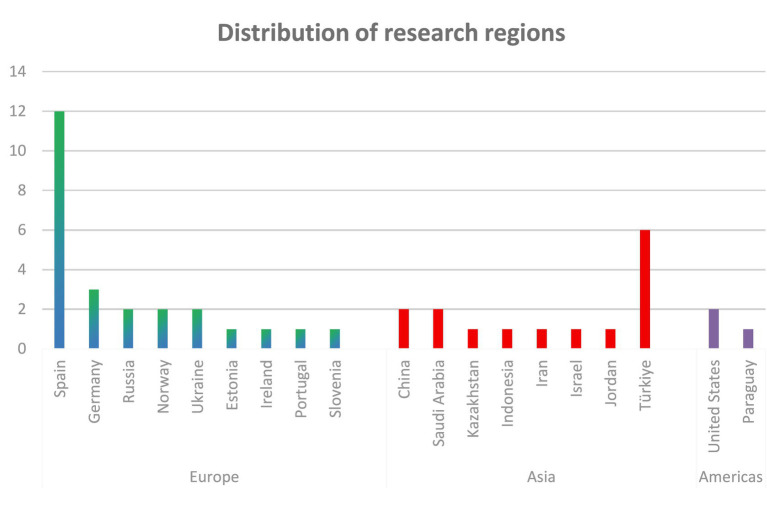
Distribution of research areas.

First, in terms of geographical distribution, the studies covered Europe (Spain, Germany, Turkey, Estonia, Norway, Slovenia, and Portugal), Asia (China, Iran, Russia, Indonesia, and Israel, Saudi Arabia and Jordan) and the Americas (the United States and Paraguay), reflecting a global interest in educational issues. Spain (12 articles) led significantly in terms of the number of studies, while other countries showed a more balanced distribution, indicating the international dispersion of research activities. The participation of Kazakhstan, Ireland and some other countries, though limited in number, broadened the geographical scope by including less-explored educational contexts. The specific regions are listed in Figure 7 (with detailed information available in Table 4).

**Figure 8:**
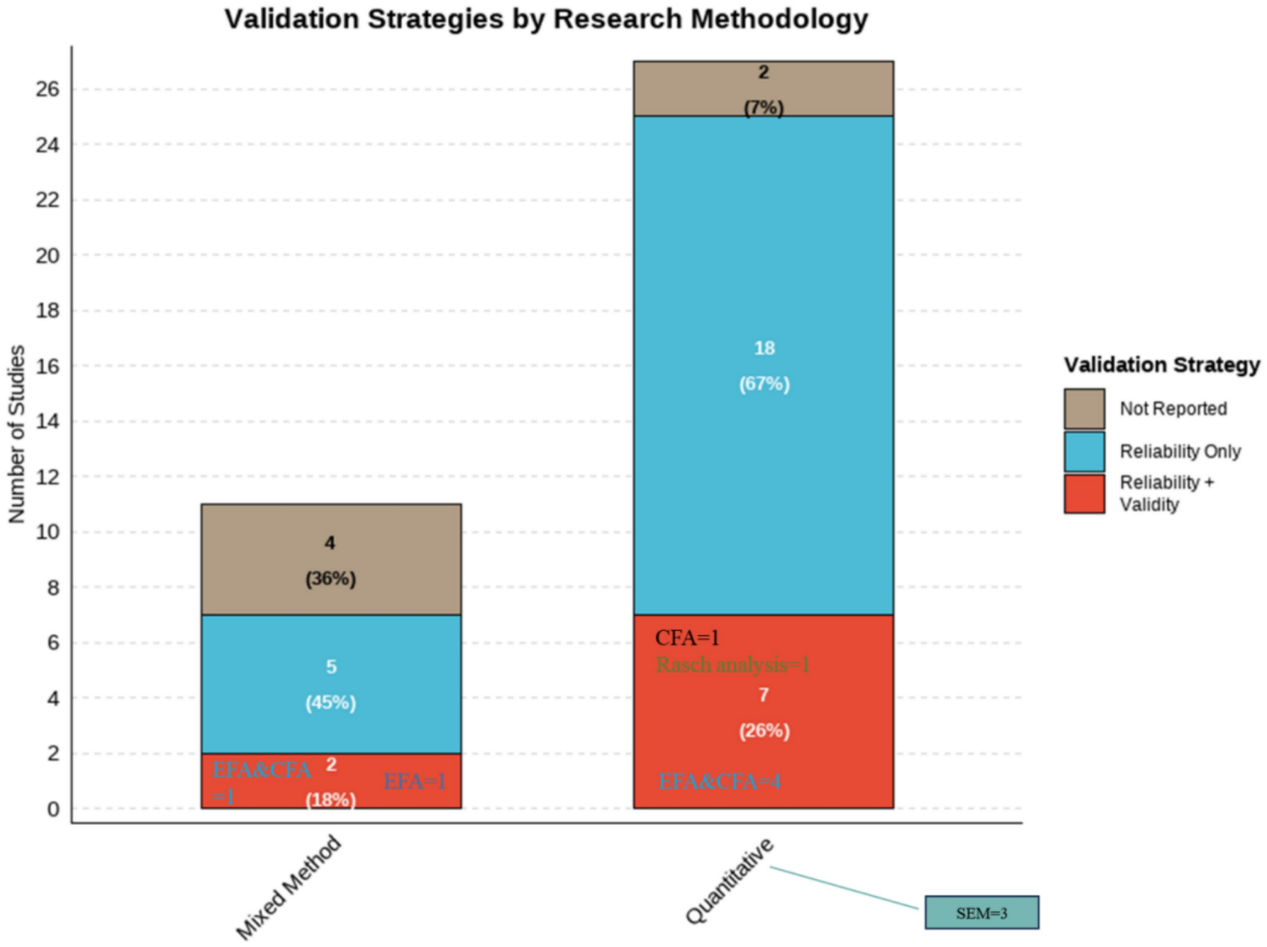
Methodology types and instrument validation counts.

Regarding methodological approaches, quantitative studies (27 articles) dominated, with 11 articles employing mixed methods, as shown in Figure 8. Detailed methodological data can be found in Table 1. Mixed-methods studies, by combining quantitative and qualitative approaches, offer a more comprehensive perspective, capturing both measurable outcomes and contextual insights that enrich the understanding of pre-service teachers’ digital competence. As Hughes et al. (2023) demonstrated, mixed methods overcome the limitations of relying solely on quantitative or qualitative research, enabling a more comprehensive understanding of the research.

Notably, the application of structural equation modeling (SEM, two articles) and partial least squares structural equation modeling (PLS-SEM, one article), though relatively limited, demonstrated researchers’ efforts to construct more sophisticated and complex theoretical models to analyze latent relationships among variables.

In terms of data collection, questionnaires and developed or validated scales were commonly used for participant self-assessment. These tools were often designed based on existing conceptual frameworks, such as the DigComp model and TPACK framework, to ensure systematic and scientific rigor. Additionally, interviews and tests were employed in some studies to supplement these questionnaires and scales. Tests directly assess participants’ digital knowledge levels and attitudes, while interviews, particularly semi-structured ones, allow for a deeper evaluation of participants’ digital competence. Some studies also collected participants’ diaries, included open-ended questions in questionnaires, or had trainers periodically consult participants about their competencies.

In the tool validation phase, most studies primarily reported Cronbach’s *α* for reliability assessment, with formal validity verification (e.g., EFA, CFA) rarely conducted across both quantitative and mixed-method studies (as shown in Figure 8). Specifically, among quantitative studies, 18 (67%) only reported Cronbach’s *α*, 7 (26%) verified both reliability and validity (including one applying Rasch analysis), and 2 (7%) provided no validation information. For mixed-method studies, the proportion of studies that provided no validation details was notably higher: 4 (36%) did not report any validation information, 5 (45%) only addressed reliability via Cronbach’s *α*, while merely 2 (18%) conducted both reliability and validity checks.

### 4.5 Limitations of the selected studies

Reviewing these studies reveals that research on pre-service teachers’ digital competence is constrained by limitations in sample representativeness, data collection instruments, research design, contextual diversity, and the range of variables examined (see Table 5; detailed information on the relevant articles is available in Table 1).

**Table 5:** The main limitations of the selected articles.

Limitation category	Number of studies	Percentage of total studies (38)	Key issues
Data collection tools	25	65.79%	Reliance on self-reported data; lack of diversified methods; inadequate validation
Sample representativeness	23	60.53%	Small sample sizes; limited to specific regions or groups
Research content/variables	14	36.84%	Narrow scope of variables; insufficient dimensions
Research design	8	21.05%	Cross-sectional designs; lack of experimental designs
Research context/environment	4	10.53%	Impact of the COVID-19 pandemic

First, limitations in data collection tools (25/38) are particularly notable. Some studies rely on self-reported data, which may introduce subjective biases (Luik et al., 2018), and some lack diversified methods of data collection.

Second, insufficient sample representativeness (23/38) is a major issue, as many studies rely on small sample sizes (Belda-Medina and Calvo-Ferrer, 2022; Miguel-Revilla et al., 2020) or are limited to specific regions or groups (Yang et al., 2022; Hairida et al., 2023), whichmay limit the generalizability of the findings. Furthermore, limitations in research content and variables (14/38) are evident in the narrow scope of variables and insufficient dimensions, failing to comprehensively assess all key areas of digital competence (Andreasen et al., 2022).

Moreover, limitations in research design (8/38) also affect the reliability of the studies. For example, cross-sectional designs cannot capture changes in competence over time (Galindo-Domínguez and Bezanilla, 2021), and the absence of experimental designs makes establishing causal relationships difficult.

Finally, limitations in research context and environment (4/38), such as the impact of the COVID-19 pandemic (Torres-Hernández and Gallego-Arrufat, 2024; Quast et al., 2023), may have influenced results, reducing their broader applicability.

## 5 Discussion

### 5.1 Main trends, frameworks, and research focuses

The annual publication trend of the selected studies on pre-service teachers’ digital competence from 2014 to 2024 shows a significant upward trajectory, reflecting the growing societal attention to digital technologies, catalyzed by the successive release of European frameworks (DigCompEdu, INTEF), and further accelerated after 2018 by rapid educational informatisation and the COVID-19 pandemic (Díaz-Burgos et al., 2024). At the same time, the expanding application of TPACK, DigCompEdu and AI-TPACK has shifted the research focus from “do pre-service teachers possess digital literacy?” to “are they ready for online or AI-enhanced pedagogy?,” reaffirming that technological knowledge gains significance only when integrated with pedagogical and content contexts.

Significant disparities exist in the number of research articles across different regions, indicating regional concentrations and an imbalance in research activities. European countries show more active engagement in related research, potentially due to the European Union’ s early adoption of digital transformation policies. Regions with limited research may be in the initial stages of exploration in this field or may have relatively limited research resources. In the future, enhanced international collaboration and resource sharing could promote more balanced and in-depth research in this field.

In terms of conceptual and assessment frameworks, the reviewed studies demonstrate considerable diversity. Researchers have drawn on international standards, regional policies, and author-developed models to define and assess digital competence. However, this diversity also led to several issues, as the different frameworks exhibit variations and overlaps (Torres-Hernández and Gallego-Arrufat, 2024), making the integration of these frameworks into a unified teaching or training system a challenging task.

Among the most widely used models, TPACK provides a macro-level theoretical lens, offering strategic guidance on when and how to employ technology to enhance pedagogical effectiveness (Luik et al., 2018; Miguel-Revilla et al., 2020). In contrast, DigComp framework outlines micro-level dimensions of digital competence (Alnasib, 2023; Çebi and Reisoğlu, 2020), equipping teachers with specific operational skills such as information management, content creation, and problem solving. The integration of these frameworks, as demonstrated in Yang (2024), enriches both theoretical and practical understanding while addressing the limitations of single-model approaches. Because each framework highlights distinct yet complementary aspects of digital competence, their integration has emerged as an important research direction. However, the path to achieving this integration is not without challenges. A central difficulty lies in reconciling their divergent assessment criteria and indicators, which can result in inconsistent evaluations of digital competence. Future research should focus on cross-theoretical dialogue to identify context-specific integration strategies through empirical validation, maximizing theoretical diversity’s role in pre-service teacher education.

Regarding research foci, these studies mainly focused on several key areas, including the assessment of competence levels and their influencing factors, the evaluation of training effectiveness, the validation of frameworks and assessment tools, and the investigation of the application of specific digital technologies. Overall, this field has gradually evolved from descriptive analyses of competence levels toward more application-oriented inquiries that highlight training design, assessment development, and technology integration.

Across studies, a pattern of uneven performance emerges among competence dimensions (Çebi and Reisoğlu, 2020; Llanes, 2023; Rahimi and Mosalli, 2024). Most pre-service teachers demonstrate sufficient digital skills for daily needs, but some of them may show weaknesses in digital content creation and pedagogical integration (Yang et al., 2022). Some studies based on DigComp or DigCompEdu-frameworks report Digital Content Creation as low (Llanes, 2023; Çebi and Reisoğlu, 2020) and communication and collaboration as high (Jiménez-Hernández et al., 2020; Çebi and Reisoğlu, 2020). Cross-country comparisons reveal contextual differences. For instance, problem-solving competence tends to be low in Turkey but high in Spain, possibly due to Turkey’s theory-oriented curriculum versus Spain’s hands-on telecollaboration projects (Çebi and Reisoğlu, 2020; Llanes, 2023).

A recurrent finding is that pre-service teachers often struggle to effectively translate acquired digital skills into teaching practice, largely due to limited opportunities for authentic teaching practice during their training, resulting in a disconnect between their digital technology knowledge and practical application in real teaching scenarios. For example, studies grounded in the TPACK framework repeatedly report that pre-service teachers exhibited consistently low TPK integration levels (Belda-Medina and Calvo-Ferrer, 2022; Luik et al., 2018; Miguel-Revilla et al., 2020). However, systematic interventions, like project-based learning, SQD strategies, digital storytelling, etc., could result in enhancement of TPK; suggesting that well-designed, practice-oriented activities are crucial for competence development (Çebi et al., 2022; Miguel-Revilla et al., 2020; Kukul, 2022; Hirsch and Rubach, 2024). Similar findings have emerged from research on in-service teachers (Wheeler et al., 2012), indicating a widespread issue across the teaching profession regarding the application of technology to complex operations and teaching practices, which requires systematic training and practical opportunities.

Evidence from INTEF-aligned studies further confirms this imbalance. Studies show that participants in both INTEF-aligned programs were confident in finding information and sharing files, but hesitated to create or edit digital materials before training (Galindo-Domínguez and Bezanilla, 2021; Hijón-Neira et al., 2023). This pattern persisted after the interventions: information retrieval and digital communication—maintained as relative strengths—still received higher ratings, while content creation and problem solving, despite notable gains, remained comparatively weak. The two datasets therefore converge in underscoring the need for extended, authentic creation-centered courses and activities for addressing technology-related difficulties in teaching practice to promote balanced development across all five dimensions.

In terms of factors, pre-service teachers’ digital competence is influenced by a combination of demographic, psychological, educational, and socio-cultural factors. Personal background factors such as gender, age, educational background, and learning experiences do not influence pre-service teachers’ digital competence in isolation. Instead, these variables interact in complex ways and are further intertwined with psychological attributes such as confidence and attitudes. While gender and age may independently exhibit significant correlations with digital skills, their explanatory power for digital competence is substantially enhanced when considered alongside psychological factors. This may be due to the mediating role of confidence, which interacts with background factors to shape digital ability. This highlights the need for teacher education programs to provide holistic support that develops both digital skills and the confidence to apply them effectively in teaching.

### 5.2 Methodological characteristics and limitations

Among the included studies, three research methods were identified: quantitative research, mixed methods, and structural equation modeling (SEM). Quantitative research dominated in terms of article count, indicating its popularity in academic research. This may be because quantitative research provides objective, quantifiable data that facilitate comparisons and verification within the academic community. Although less prevalent than quantitative research, mixed-method research has been applied in some complex research scenarios. By combining the strengths of quantitative and qualitative approaches, mixed method research not only provides objective and accurate data support but also enables in-depth exploration of underlying causes and mechanisms. However, SEM is used least frequently, possibly due to its high requirements for data quality, sample size, and statistical expertise, though it offers unique advantages in handling complex variable relationships and validating theoretical models. Notably, multiple authors have emphasized that different research methods are not mutually exclusive but rather complementary. For complex research questions, single research methods often prove insufficient, and thus, combining multiple methods can provide a more comprehensive and nuanced understanding (Hirsch and Rubach, 2024; Andreasen et al., 2022).

However, the reliability of research conclusions depends not only on methodological choices but also on implementation rigor. While a subset of the included studies has acknowledged the importance of instrument validation, only a fraction have actually employed rigorous psychometric techniques (such as EFA/CFA, or Rasch analysis) to validate their measurement tools. This limitation is of particular concern, as it undermines the reliability and validity of aggregated findings across studies. Among the two representative TPACK framework-based studies included in this review, the Estonian study (Luik et al., 2018) conducted systematic scale validation via Exploratory Factor Analysis (EFA) and Confirmatory Factor Analysis (CFA), while the Spanish study (Belda-Medina and Calvo-Ferrer, 2022) lacked rigorous validation and only referenced the scale source.

This validation difference may directly impact result reliability, leading to conflicting findings between the two studies: the Estonian study concluded that technological integration knowledge (TPACK core cross-dimension) is strongest while pedagogical knowledge (PK) is weakest. Conversely, the Spanish study reached the contradictory finding that TPACK core cross-dimensions are weaker than foundational dimensions. Such inconsistencies further undermine the robustness of aggregated conclusions in systematic reviews, as unvalidated tools introduce random bias, making it difficult to determine whether cross-study differences reflect real competency variations or measurement errors. To address these challenges, future research should establish standardized validation procedures, such as mandatory EFA, Cronbach’s *α* ≥ 0.7, context-specific scale adaptation, and transparent reporting of validation metrics to enhance measurement accuracy, result credibility, and cross-study comparability.

Finally, several key limitations have been identified, including insufficient sample representativeness, less rigorous research designs, interference from background factors, and incomplete variable coverage. These limitations collectively affect the accuracy and generalizability of the research findings, restricting our comprehensive understanding of pre-service teachers’ digital competence. Particular attention should be paid to the validity of self-assessment methods, as recent studies indicate a significant tendency toward overestimation among participants in their self-evaluations of competence (McGarr and McDonagh, 2021; Scherer et al., 2017), revealing a notable gap between subjective assessments and actual performance (Tomczyk et al., 2023).

This cognitive bias, known as the Dunning-Kruger effect, both hinders professional growth by preventing less competent individuals from recognizing their limitations and generates specific challenges for digital competence research. For one, it risks painting an overly optimistic picture of current digital competence levels in our aggregated findings. For instance, while many self-report studies suggest pre-service teachers have satisfactory digital skills, in reality, they often lack the practical ability in key dimensions like digital content creation or Technological Pedagogical Knowledge (TPK) integration (Çebi and Reisoğlu, 2020; Belda-Medina and Calvo-Ferrer, 2022). For another, this bias might lead to the overestimation of training intervention effectiveness as the psychological boost from participation in training may inflate pre-service teachers’ self-assessments, making it difficult to distinguish genuine competence gains from subjective bias.

The problem is further compounded by insufficient sample representativeness, as limited sample sizes, restricted demographic coverage, or contextual constraints may collectively compromise the generalizability and inferential validity of findings regarding key variables. For instance, two small-scale studies, one in Spain (Galindo-Domínguez and Bezanilla, 2021) with 200 pre-service teachers and another in Saudi Arabia (Al-Abdullatif, 2019) with 113 participants, converged in finding no statistically significant effects of gender, age, or university type on digital competence, ICT use, technical knowledge, or TPACK confidence. These findings contrast with those of larger-scale research included in our review; for instance, Torres-Hernández and Gallego-Arrufat (2024), in a nationwide survey of 1,366 pre-service teachers in Spain, identified gender, age, and degree program as significant differentiating factors across multiple dimensions of digital competence. Although, such discrepancies are not attributable to a single cause, smaller samples (typically ≤ 200 participants) often lack sufficient statistical power to detect subtle variable relationships, a limitation that may be compounded by sampling biases or narrow demographic scope. These constraints can lead to overly simplistic conclusions about the factors shaping pre-service teachers’ digital competence.

Together, these limitations underscore the need for more objective, representative, and methodologically rigorous measures in future research, including larger and more diverse samples, validated assessment tools, and careful consideration of confounding variables.

## 6 Conclusion

This systematic review analyzes research on pre-service teachers’ digital competence (2014–2024), examining trends, conceptual frameworks, research focuses, while also accounting for regional disparities and methodological limitations. The findings reveal gaps in complex technical operations and teaching applications, as well as limitations in current training programs regarding technology integration and innovation.

This study highlights the societal importance of improving pre-service teachers’ digital competence to enhance educational quality and equity, thereby supporting sustainable development in the digital era. By equipping teachers with the ability to effectively use digital technologies, this work contributes to high-quality education and long-term societal progress.

This study also offers actionable, sustainable, and scalable insights for teacher training institutions and education policymakers. For teacher training institutions, assessment systems should be refined as dynamic frameworks rather than static tools: indicators need regular updates aligned with teaching contexts, subject features, and digital technology advancements (e.g., integrating AI-assisted teaching metrics as new tools emerge), while incorporating process-oriented metrics such as training participation rate. In this regard, development of a standardized, adaptable assessment toolkit, including case analysis guidelines, simulated teaching evaluation criteria, and peer feedback forms, for sharing across institutions would facilitate sharing and consistent implementation across institutions. In training practice, key challenges should be addressed within compulsory courses by incorporating sustained micro-teaching sessions with immediate mentor feedback and requiring each pre-service teacher to design, implement, and revise at least one technology-infused lesson plan integrating digital tools, pedagogy, and subject content.

For ministries and education policymakers, formalizing these practices into national standards and funding mechanisms is key to scalability. First, allocate earmarked funds for advanced digital classrooms and mentor training at teacher education institutions, with a tiered funding model to accommodate different levels, ensuring equitable access. Second, integrate ongoing tech-integration training into national teacher qualification standards and in-service teacher professional development requirements, mandating periodic recertification to sustain competence. Building on these policies, cross-institutional collaboration should be promoted to validate assessment tools across diverse cultural and educational settings, building a shared database of validated metrics to ensure reliability and reduce redundant development efforts.

Despite offering valuable insights for research and practice, this review has some limitations that should be noted. First, it only included studies from Web of Science and Scopus and used keywords limited to “digital competence,” “digital literacy,” and “digital skill,” excluding ICT-related research. This may have omitted relevant studies. Future studies should include other databases and keywords, including ICT terms.

Second, the methodological choice of excluding purely qualitative studies potentially introduces geographical representativeness bias. Qualitative research dominates in Nordic countries (e.g., 52.39% of studies in Sweden adopt a qualitative design) (Skau et al., 2025) and African regions (e.g., Bladergroen and Chigona, 2018; van Wyk and Waghid, 2023). The exclusion of these studies renders it difficult for this review to fully present the diverse geographical landscape of global research on pre-service teachers’ digital competencies. Third, this review included only English-language publications, limiting its global applicability. Incorporating multilingual studies would enhance its representativeness.

Future research should address these limitations by employing larger and more diverse samples, integrating mixed-methods designs, and including multilingual and qualitative studies. It could also explore digital competence in specific subjects or grade levels and examine in-service teachers’ technology use and pre-service preparation effectiveness to inform improvements to teacher education.

## Data Availability

The original contributions presented in the study are included in the article/supplementary material, further inquiries can be directed to the corresponding author.
